# Allophycocyanin A is a carbon dioxide receptor in the cyanobacterial phycobilisome

**DOI:** 10.1038/s41467-022-32925-6

**Published:** 2022-09-08

**Authors:** Alejandra Guillén-García, Savannah E. R. Gibson, Caleb J. C. Jordan, Venkata K. Ramaswamy, Victoria L. Linthwaite, Elizabeth H. C. Bromley, Adrian P. Brown, David R. W. Hodgson, Tim R. Blower, Jan R. R. Verlet, Matteo T. Degiacomi, Lars-Olof Pålsson, Martin J. Cann

**Affiliations:** 1 Department of Biosciences, Biophysical Sciences Institute, Durham University, South Road, Durham, DH1 3LE UK; 2 Department of Chemistry, Biophysical Sciences Institute, Durham University, South Road, Durham, DH1 3LE UK; 3 Department of Physics, Biophysical Sciences Institute, Durham University, South Road, Durham, DH1 3LE UK; 4 Biophysical Sciences Institute, Durham University, South Road, Durham, DH1 3LE UK

**Keywords:** Post-translational modifications, Proteins, Bacterial physiology, Photosynthesis

## Abstract

Light harvesting is fundamental for production of ATP and reducing equivalents for CO_2_ fixation during photosynthesis. However, electronic energy transfer (EET) through a photosystem can harm the photosynthetic apparatus when not balanced with CO_2_. Here, we show that CO_2_ binding to the light-harvesting complex modulates EET in photosynthetic cyanobacteria. More specifically, CO_2_ binding to the allophycocyanin alpha subunit of the light-harvesting complex regulates EET and its fluorescence quantum yield in the cyanobacterium *Synechocystis* sp. PCC 6803. CO_2_ binding decreases the inter-chromophore distance in the allophycocyanin trimer. The result is enhanced EET in vitro and in live cells. Our work identifies a direct target for CO_2_ in the cyanobacterial light-harvesting apparatus and provides insights into photosynthesis regulation.

## Abstract (web summary)

The transfer of electronic energy through a photosystem can harm the photosynthetic apparatus when not balanced with CO_2_ fixation. Here, the authors show that CO_2_ modulates electronic energy transfer in cyanobacteria by binding to and enhancing the activity of the light-harvesting complex.

## Introduction

Oxygenic photosynthesis evolved in the cyanobacteria about 2.5 Gyr ago and provided much of our current atmospheric O_2_^1^. Cyanobacteria gather photons using a harvesting antenna known as the phycobilisome (PBS). Phycobiliproteins (PBPs) constitute PBSs to produce one of the largest protein complexes known. The PBPs include phycocyanin, phycoerythrin and allophycocyanin, together with their associated linker proteins^2^. PBS light absorption is achieved by open-chain tetrapyrrole (bilin) chromophores covalently attached to the PBPs and their associated linker proteins^3^. Two subunits of a PBP, an α- and a β-subunit, form an αβ heterodimer known as an (αβ) monomer, which is assembled into an (αβ)_3_ trimer. Electronic energy transfer (EET) within the (αβ)_3_ trimer is fast and efficient (on the sub-ps to ps timescales)^4^. The PBP (αβ)_3_ trimers form the ordered supramolecular light-harvesting complex via the linker proteins^5^. A typical cyanobacterial hemidiscoidal PBS consists of a central core surrounded by peripheral chromophore-containing rods that capture photons^6^. The PBS core transfers excitation energy via a terminal emitter to the photosynthetic reaction centres with the subsequent synthesis of ATP and NADPH^7^.

CO_2_ assimilation into sugars requires NADPH and ATP. There is evidence from non-cyanobacterial model systems that CO_2_ fixation and availability are coupled to light-harvesting^8,9^. For example, CO_2_-deficient conditions in algae reduce photosystem (PS) II antenna size, and PSI/PSII fluorescence intensity increases^10,11^. A light-harvesting chlorophyll–protein complex II migrated from PSII to PSI under CO_2_-deficient conditions in *Chlamydomonas*^12^. Insufficient CO_2_ can also result in over-reduction of the plastoquinone pool, resulting in singlet oxygen species and photoinhibition^13^. The addition of a suitable carbon source under CO_2_-deficient conditions restored photosynthetic activity^14^.

The mechanism(s) that couples inorganic carbon availability to light-harvesting is uncertain. In higher plants, bicarbonate binding to PSII provides a redox tuning mechanism regulating and protecting PSII^15^. However, questions remain as HCO_3_^−^ is also proposed to be tightly bound to PSII under physiologically relevant environmental CO_2_, meaning this binding site’s role as an authentic regulatory site at limiting CO_2_ awaits further experimentation^16^. Nevertheless, there is considerable evidence that CO_2_/HCO_3_^−^ availability can regulate light-harvesting and subsequent EET.

How might CO_2_ regulate light-harvesting? CO_2_ and protein can interact through carbamates on neutral *N*-terminal α-amino- or lysine ε-amino groups. For example, carbamylation regulates the activities of Rubisco^17^, haemoglobin (Hb)^18^, and ubiquitin^19^. In addition, several proteins carry a stable carbamate required for catalysis, e.g., urease, alanine racemase, transcarboxylase 5 S, class D β-lactamase and phosphotriesterase^20^. Therefore, we and others have hypothesized that reversible carbamylation of neutral *N*-terminal α-amino groups and/or lysine ε-amino groups could form a widespread mechanism for protein regulation by CO_2_^17,20^. We developed triethyloxonium tetrafluoroborate (TEO) as a chemical proteomics tool to identify carbamate post-translational modifications (PTMs)^20^. We hypothesised the TEO chemical proteomics tool could be used to discover CO_2_-binding proteins in the cyanobacterial PBS to determine the mechanistic basis for coupling CO_2_ availability to EET. Here we describe the discovery of a CO_2_-binding site in the PBS and its role in regulating EET.

## Results

### *Synechocystis* sp. PCC 6803 allophycocyanin A binds CO_2_

**Fig. 1:**
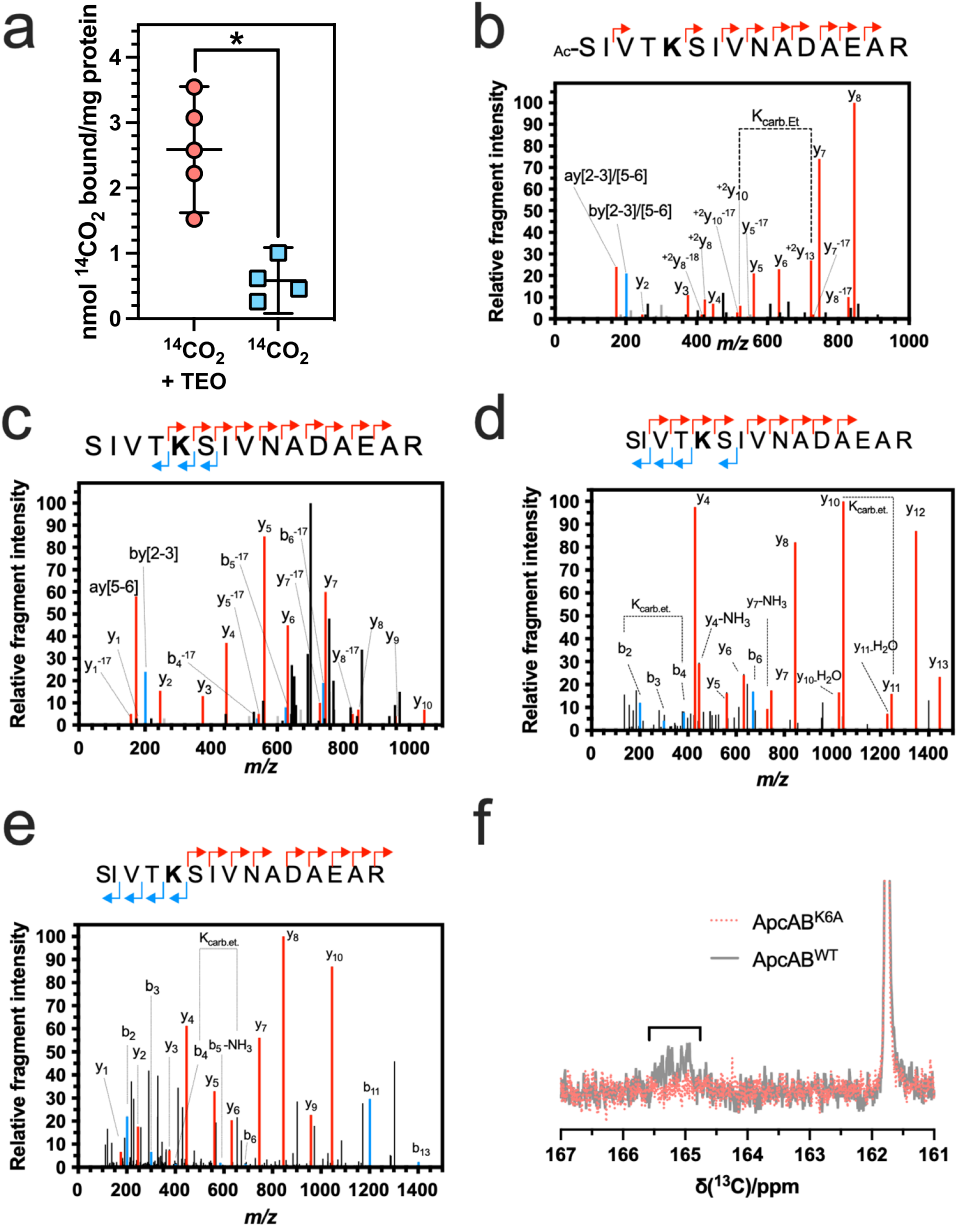
CO_2_ binds ApcA. **a** Demonstration of exchangeable CO_2_-binding sites on *Synechocystis* protein extract. CO_2_ trapped onto protein extract of *Synechocystis* sp. PCC 6803 (*p = 0.002, two-tailed *t*-test, *n* = 5 independent replicates for ^14^CO_2_ + TEO and *n* = 4 independent replicates for ^14^CO_2_ only, *t* = 4.794, *df* = 7, mean ± 95% CI). **b**–**e** Demonstration of exchangeable CO_2_-binding sites on ApcA by MS/MS. Plots of relative fragment intensity versus mass/charge ratio (m/z) for fragmentation data from MS/MS identifying ethyl-trapped carbamate on whole *Synechocystis* sp. PCC 6803 (**b**), isolated phycobilisomes from *Synechocystis* sp. PCC 6803 (**c**) or recombinant *Synechococcus* sp. PCC 7002 ApcAB^WT^ (αβ)_3_ trimers (**d**, **e**) in the presence of ^12^CO_2_ (**b**–**d**) or ^13^CO_2_ (**e**). Peptide sequences indicate predominant +1 y (red) +1b (blue) ions identified by MS/MS shown in the plot. Other observed ions, not labelled on the figure panels for clarity, are shown in grey and identified in Supplementary Table 1 (whole *Synechocystis* sp. PCC 6803) and Supplementary Table 3 (isolated phycobilisomes from *Synechocystis* sp. PCC 6803). The modified residue is indicated in bold. K_carb.Et_ indicates the molecular weight difference between ions diagnostic of the modified Lys. **f** ^13^C-NMR spectrum demonstrating the formation of a carbamate on recombinant *Synechococcus* sp. PCC 7002 ApcAB^WT^ (αβ)_3_, but not the mutant ApcAB^K6A^ (αβ)_3_, trimers by the appearance of a peak at 165 ppm. Source data are provided as a Source Data file.

An extract of *Synechocystis* sp. PCC 6803 soluble protein was incubated with 20 mM NaH^14^CO_3_ and subjected to TEO-trapping (Fig. 1a). An insignificant level of ^14^CO_2_ was incorporated into the protein extract in the absence of TEO. The inability to observe protein-bound ^14^CO_2_ in the absence of TEO is likely due to the ready reversibility of carbamylation that leads to sample degassing during preparation for analysis. However, the TEO-trapped protein lysate contained significant protein-bound ^14^CO_2_, even when accounting for 50% of the total protein sample being Rubisco. Thus, we conclude that the *Synechocystis* proteome contains CO_2_-interacting proteins carbamylated at labile sites exchangeable with the environment. We proceeded to identify carbamate PTMs to investigate proteins that couple CO_2_-binding to EET.

Soluble *Synechocystis* protein lysate was equilibrated with 20 mM CO_2_/HCO_3_^−^ at pH 7.4, and TEO was added to trap carbamate PTMs. The trapping reaction mixture was digested with trypsin, and samples were analysed by ESI-MS/MS (Electrospray Ionisation Tandem Mass Spectrometry). The data were interrogated for variable PTMs on lysine with masses of 72.0211 Da (trapped carbamate) and 28.0313 Da (*O*-ethylation on glutamate and aspartate side chains). A lysine carbamylation site was identified on open reading frame Slr2067 (MS/MS peptide amino acids 2–16 SIVT**K**SIVNADAEAR, proposed carbamylation on K6) (Fig. 1b; Supplementary Fig. 1a; Supplementary Table 1). The MSMS spectrum lacked detectable b ions crossing the modification. Therefore, independent experiments were performed and identified the same carbamylation site with either both y and b ions or only b ions crossing the modification (Supplementary Fig. 2; Supplementary Table 2). In all cases, the K6 carbamate was identified on the internal lysine residue of the peptide, a so-called missed cleavage. Carbamylation removes the positive charge on the lysine that is essential for cleavage site recognition by trypsin. This missed cleavage supports the Slr2067 K6 carbamate as genuine as a missed cleavage is an otherwise rare event, despite the suboptimal spectra quality. The slr2067 gene encodes the allophycocyanin α-subunit (ApcA). ApcA is the α-subunit of the allophycocyanin (αβ)_3_ trimer located within the PBS core. It has a critical role in EET from the PBS antennal proteins to the photosystem via the terminal pigment. We identified the same carbamate PTM on ApcA residue K6 in TEO-trapping experiments performed on isolated PBS (Fig. 1c; Supplementary Table 3) and purified recombinant ApcAB (αβ)_3_ trimer (Fig. 1d; Supplementary Fig. 3). We performed the CO_2_ trapping experiments on ApcA with ^13^CO_2_ to corroborate the carbamate PTM by interrogating the MS/MS data for a 73.0211 Da modification. The expected +1 Da m/z increase (from 72.0211 Da) was observed for the carbamylation site at K6 (Fig. 1e). We performed a control experiment to identify the reason for the relative difficulty in observing multiple ions that encompass the K6 carbamate. Synthetic SIVTKSIVNADAEAR peptide was equilibrated with 50 mM CO_2_/HCO_3_^−^ at pH 8.5, TEO was added, and the peptide analysed by MS/MS (Supplementary Fig. 4; Supplementary Tables 4–6)^20^. MS/MS fragment ions that cross K6 were observed to be lower intensity for the carboxyethylated peptide compared to the unmodified or ethylated peptide (note how the intensity of the labelled ions is almost indistinguishable from background non-specific ions in Supplementary Fig. 4b compared to Supplementary Fig. 4a, c). Therefore, the relative difficulty of observing the carbamate modification on K6 by LC-MS/MS is an intrinsic property of the carboxyethylated peptide.

To compensate for the relative difficulty in using MS/MS to observe a carbamate at K6, we used ^13^C-NMR as an orthologous method to confirm the ApcA CO_2_-binding site at K6. Carbamate formation on wild type recombinant ApcA (αβ)_3_ trimer (ApcAB^WT^ (αβ)_3_) was confirmed using ^13^C-NMR spectroscopy by observing a signal at ~165 ppm, which matched literature values for carbamate formation (Fig. 1f)^21^. We generated a K6A mutant recombinant protein (ApcAB^K6A^ (αβ)_3_) to investigate whether the observed ^13^C-NMR signal was due to the carbamate forming at K6. We also generated a K6E mutant recombinant protein (ApcAB^K6E^ (αβ)_3_) as we hypothesised that mutation of K6 to glutamate would represent the local carbamate charge state at 100% occupancy. Circular dichroism spectra for ApcAB^WT^ (αβ)_3_ versus ApcAB^K6A^ (αβ)_3_ and ApcAB^K6E^ proteins demonstrated that the three proteins possessed similar secondary structures (Supplementary Fig. 5). Therefore, there is no gross alteration in the ApcAB^K6A^ (αβ)_3_ structure in the mutant protein. No signal corresponding to a carbamate was observed in ApcAB^K6A^ (αβ)_3_ trimers. ApcA is, consequently, a CO_2_-binding protein via carbamylation of K6 at photosynthetically appropriate gas levels^22^.

Carbamylated ApcA peptide detection was not sufficiently reliable to quantify carbamylation at varying CO_2_/HCO_3_^−^ by either labelling or label-free methods and ^13^C-NMR was insufficiently sensitive. However, we hypothesised that increased ApcA carbamylation could be indirectly monitored through a decreased observation of the unmodified peptide. ApcAB^WT^ (αβ)_3_was equilibrated with varying CO_2_/HCO_3_^−^ at pH 7.4, TEO was added to trap carbamate PTMs, and samples were analysed by ESI-MS/MS. However, the unmodified K6-containing peptide was more reliably detected by MS/MS than the modified peptide. Therefore, we measured the peak area of the unmodified K6-containing peptide and calculated it as a ratio of the peak area of four other control peptides. At varying CO_2_/HCO_3_^−^, this ratio was compared to the absence of CO_2_/HCO_3_^−^ (Supplementary Fig. 6). The control peptides were also compared to one another. CO_2_/HCO_3_^−^ caused an immediate decrease in the relative amount of the unmodified peptide, which we infer is due to an increase in the modified peptide. This decrease did not occur for the control peptides. The equivalent drop in the K6-containing peptide at all [CO_2_/HCO_3_^−^] suggested that the carbamate might be acting as an on/off switch in the presence or absence of CO_2_.

### Allophycocyanin A CO_2_-binding regulates EET in vitro

**Fig. 2:**
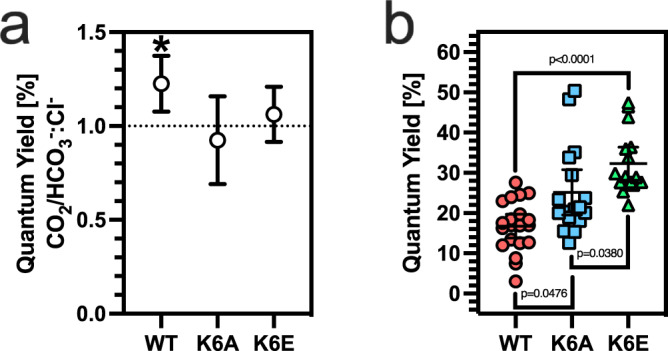
CO_2_ enhances ApcAB^WT^ (αβ)_3_ QY in vitro. **a** Ratio of the measured fluorescence QY with 20 mM CO_2_/HCO_3_^−^ or NaCl for recombinant *Synechococcus* sp. PCC 7002 ApcAB^WT^ (αβ)_3_, ApcAB^K6A^ (αβ)_3_, or ApcAB^K6E^ (αβ)_3_ trimers (*p = 0.0036, one sample *t*-test, ratio is derived from *n* = 11 independent replicates for WT and *n* = 7 independent replicates for K6A, mean ± 95% CI). **b** Basal fluorescence QY for recombinant *Synechococcus* sp. PCC 7002 ApcAB^WT^ (αβ)_3_, ApcAB^K6A^ (αβ)_3_, or ApcAB^K6E^ (αβ)_3_ trimers (Kruskal-Wallis test, *n* = 20, 17, 16 independent replicates for WT, K6A, and K6E respectively, mean ± 95% CI). Source data are provided as a Source Data file.

We analysed the spectroscopic properties of ApcAB (αβ)_3_ trimers to gain insight into the influence of CO_2_ on EET. We first compared the excitation (Supplementary Fig. 7) and emission (Supplementary Fig. 8) spectra of ApcAB^WT^ (αβ)_3_ and ApcAB^K6A^ (αβ)_3_ trimers. CO_2_/HCO_3_^−^, but not NaCl (as a control for the Na^+^ cation), enhanced the excitation and emission spectra in wild type but not K6A mutant (αβ)_3_ trimers. Calculation of the fluorescence quantum yield (QY) provides information on the efficiency of EET through the PBS to the photosynthetic reaction centre. We measured absolute fluorescence QY using an integrating sphere fibre-coupled to a fluorimeter^23^ (λ_ex_ = 610 nm; λ_abs_ = 600–620 nm; λ_em_ = 620–750 nm). Inorganic carbon (CO_2_/HCO_3_^−^) enhanced the QY for ApcAB^WT^ (αβ)_3_ trimers by 22.6 ± 0.1% (S.D.) compared to NaCl. We next asked whether ApcA K6 carbamylation was responsible for the increase in QY in ApcAB (αβ)_3_ trimers in response to CO_2_/HCO_3_^−^. Therefore, we measured fluorescence QY for recombinant ApcAB^WT^ (αβ)_3_ versus ApcAB^K6A^ (αβ)_3_ and ApcAB^K6E^ proteins in the presence of CO_2_/HCO_3_^−^ or the corresponding NaCl concentration. Only the QY for ApcAB^WT^ (αβ)_3_ trimer was enhanced by CO_2_/HCO_3_^−^ compared to NaCl (Fig. 2a). Photosynthetically relevant CO_2_/HCO_3_^−^ levels, therefore, regulate ApcAB EET dependent on carbamylation at K6.

We next compared the ps to ns fluorescence lifetimes for ApcAB^WT^ (αβ)_3_ and ApcAB^K6A^ (αβ)_3_ for emission after any internal energy transfer (Supplementary Table 7). We observed a predominant fluorescence lifetime (in a bi-exponential decay) for ApcAB^WT^ (αβ)_3_ of 1.87 ± 0.04 ns (96.8 ± 2.8 % yield; SD) and for ApcAB^K6A^ (αβ)_3_ of 1.96 ± 0.10 ns (97.0 ± 3.3 % yield; S.D.). These observations reflect the fluorescence lifetime from the emitting state of the APC antennae^24^. Therefore, the basal fluorescence properties for ApcAB^WT^ (αβ)_3_ and ApcAB^K6A^ (αβ)_3_ trimers are broadly similar.

As we hypothesised that mutation of K6 to glutamate would represent the local carbamate charge state at 100% occupancy, we compared the basal fluorescence QY for recombinant ApcAB^WT^, ApcAB^K6A^, and ApcAB^K6E^ (αβ)_3_ trimers. The basal fluorescence QY for ApcAB^K6E^ (αβ)_3_ trimer was significantly higher than both ApcAB^WT^ and ApcAB^K6A^ (αβ)_3_ trimers (Fig. 2b). Of note, the basal fluorescence QY for ApcAB^K6A^ (αβ)_3_ trimer was also significantly higher than ApcAB^WT^ (αβ)_3_ trimer but lower than the ApcAB^K6E^ (αβ)_3_ trimer. These data further support the hypothesis that CO_2_/HCO_3_^−^ alters EET dependent on carbamylation at K6.

### CO_2_ alters allophycocyanin A inter-chromophore distance

**Fig. 3:**
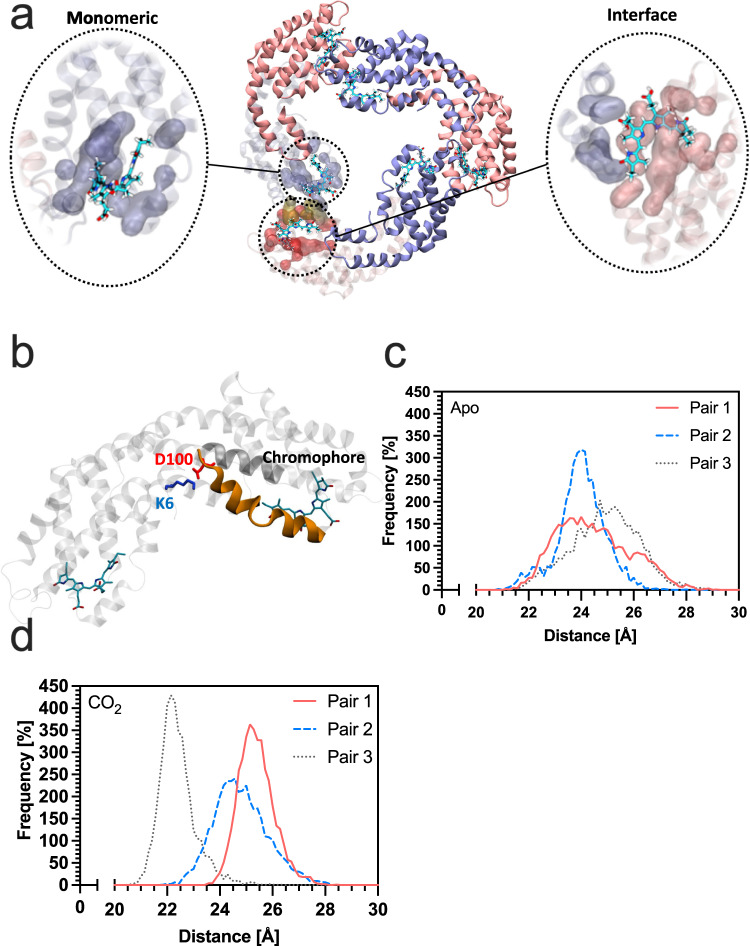
CO_2_ alters pairwise chromophore interactions in allophycocyanin trimers from *Thermosynechococcus vulcanus*. **a** Ribbon diagram of the ApcAB (αβ)_3_ trimer demonstrating the monomeric and interface chromophore-binding pockets. ApcA is shown in red and ApcB in blue. **b** K6 forms a salt bridge with D100. D100 is sited on an α-helix that connects to the chromophore-binding pocket. **c** Plot of the frequency of distance against inter-chromophore distance for three monomer-interface chromophore pairs in the apo state. **d** Plot of the frequency of distance against inter-chromophore distance for three monomer-interface chromophore pairs in the CO_2_-bound state. Source data are provided as a Source Data file.

We used molecular dynamics (MD) simulations to understand the mechanism underlying the increase in QY in ApcAB^WT^ (αβ)_3_ trimer. To this end, we performed two 1 μs simulations of *Thermosynechococcus vulcanus* (PDB: 3DBJ; ref. 25) ApcAB^WT^ (αβ)_3_ trimer (apo and CO_2_ bound). The ApcAB (αβ)_3_ trimer has six chromophore-binding pockets that we defined as either monomeric (enclosed by residues of a monomer alone) or interface (enclosed by residues of adjacent monomers) (Fig. 3a). Analysis of the root mean square deviation (RMSD) with reference to the initial state demonstrated that both simulations stabilised after 600 ns (Supplementary Fig. 9). We examined the rotamer states for K6, whether apo or CO_2_-bound (Supplementary Fig. 10, Supplementary Table 8). We observed an overall change in the occupancy of rotamer states and noted the loss of a major rotamer when CO_2_-bound corresponding to χ_1_ ~75°. Contact analysis demonstrated the disruption of a salt bridge between K6 and D100 in the CO_2_-bound state (Fig. 3b, Supplementary Fig. 11) where D100 resides on an α-helix that couples K6 to the chromophore-binding site. Two helices in the (αβ)_3_ trimer showed a higher RMSD in the CO_2_-bound state (Supplementary Fig. 12). This higher RMSD reflected a change in the contact frequency between the chromophore and binding-pocket residues for both the monomer (Supplementary Fig. 13a) and interface (Supplementary Fig. 13b) pockets as observed during the last equilibrated 300 ns of the simulations.

We asked whether the alterations in (αβ)_3_ trimer contact frequency could result in changes that explained the difference in QY with CO_2_/HCO_3_^−^. Therefore, we measured the change in distance of each chromophore pair (i.e., one chromophore bound to an interface pocket of an αβ heterodimer and one bound to a monomer pocket of another heterodimer) (Fig. 3c, d). We observed that in the last 300 ns of the simulation, distances are comparable between all three chromophore pairs (24–25 Å) without CO_2_ (Fig. 3c), but the distance of one pair reduces to 22 Å when CO_2_-bound (Fig. 3d). Therefore, the implication is that the reduced inter-chromophore separation enhances EET to the final emitting site in the system. These data would accordingly support the observations of the basal fluorescent QY for ApcAB^WT^, ApcAB^K6A^, ApcAB^K6E^ (αβ)_3_ trimers (Fig. 2c). ApcAB^K6E^ resembles a fully carbamylated ApcAB protein and thus shows the most significant increase in QY. The ApcAB^K6A^ protein also has an ablated salt-bridge between K6 and D100, so basal QY is increased relative to ApcAB^WT^ but not as much as ApcAB^K6E^ presumably because the new contacts made by the anionic carbamate are not present.

The monomer- and interface-pocket chromophores have been proposed to form exciton-coupled pairs in (αβ)_3_ trimers^26^. However, a weaker coupling regime allowing for incoherent EET according to the Förster mechanism has also been proposed^4,24^. When two chromophores with similar excitation energies are located close to one another and chirally disposed, the excited state can delocalise over both chromophores to become an exciton. Exciton interactions are important in photosynthetic light-harvesting complexes by expanding the spectral cross-section to permit faster and more robust EET^27^. However, in a weaker coupling regime, EET, according to the Förster mechanism, is applicable, and the rate of EET can be obtained for a given pairwise interaction and separation. The rate of Förster EET is provided by

$${k}_{DA}=\frac{{\kappa }^{2}}{{\tau }_{D}}{\left[\frac{{R}_{of}}{{R}_{DA}}\right]}^{6}$$

Where *R*_*of*_ and *R*_*DA*_ is the Förster radius and distance in the donor-acceptor pair, respectively. κ^2^ is a mutual orientation factor, and τ_*D*_ is the donor fluorescence lifetime. Using a Förster radius of 68.0 Å^23^, a fluorescence lifetime of 1.5 ns^24^, and a mutual orientation factor of 1.0, we obtain an EET time of 3.3 ps for a distance of 24.5 Å (without CO_2_) and 1.7 ps for a distance of 22.0 Å (in the presence of CO_2_). These values are similar to previously measured EET rates in related systems^4,24^. The orientation factor κ^2^ has a range; 0 ≤ κ^2^ ≤ 4. For κ^2^ = 1.0 the interaction dipoles adopt a “sandwich” configuration. If the orientation factor substantially impacted the rate of electronic energy transfer on CO_2_ binding, there would have to be a change in mutual dipole orientation from the “sandwich” towards an “in line” configuration. No structural change consistent with this configuration change was evidenced from the molecular dynamics simulations. Therefore, altered change in distance between interacting chromophores is the significant contributor to the modulation of the electronic energy transfer. The increased EET rate calculated from distances measured in our MD simulations correlates with the higher QY measured for ApcAB (αβ)_3_ trimers in the presence of CO_2_ and corroborates our hypothesis.

**Fig. 4:**
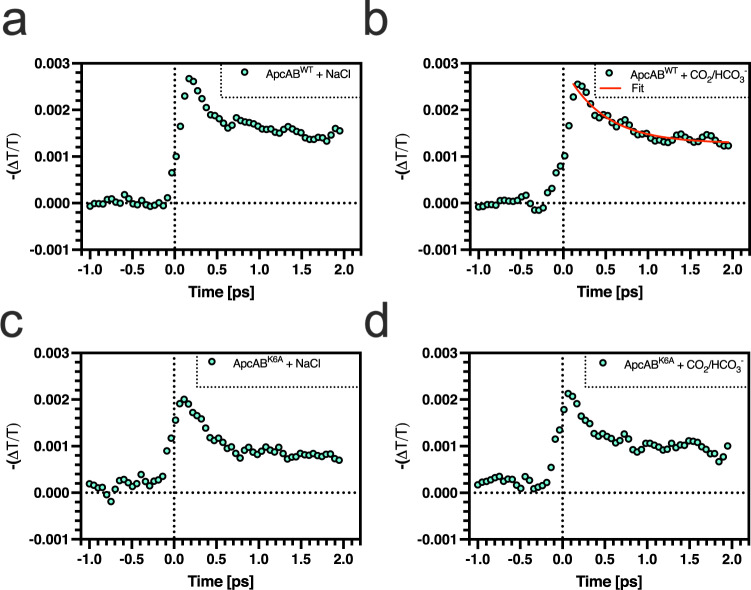
CO_2_ enhances the *Synechococcus* sp. PCC 7002 ApcAB EET rate in vitro. A plot of the change in transmission versus time for ApcAB^WT^ (αβ)_3_ (**a**, **b**) or ApcAB^K6A^ (αβ)_3_ (**c**, **d**) trimers in the presence of NaCl (**a**, **c**) or CO_2_/HCO_3_^−^. The sample was excited at 0 ps with a pulse temporal width of 200 fs at 515 nm, and transmission was analysed over the range 610–630 nm. The red line indicates the data fit for calculating EET rates. Source data are provided as a Source Data file.

We employed ultrafast transient absorption spectroscopy to validate the calculated EET rates experimentally. The ApcAB (αβ)_3_ trimers *of Synechococcus* sp. PCC 7002 used for in vitro biochemistry are very similar in structure to the ApcAB (αβ)_3_ trimers of *Thermosynechococcus vulcanus* used for molecular dynamics (Supplementary Fig. 14). *Synechoccus* ApcAB (αβ)_3_ trimers were, therefore, used for ultrafast transient absorption spectroscopy. ApcAB (αβ)_3_ trimers were monitored over a 2.5 ps time window after excitation by a ~200 fs optical pulse at 515 nm (Fig. 4, Supplementary Fig. 15). Probing the absorption recovery at 610–630 nm revealed a fast initial stimulated emission component of 200–400 fs, observed in both the absence and presence of CO_2_ in both ApcAB^WT^ (αβ)_3_ and ApcAB^K6A^ (αβ)_3_ trimers. We attribute this fast initial stimulated emission component to relaxation within the strongly coupled αβ dimer, consistent with earlier work^28^.

The 200–400 fs component is followed by a stimulated emission component in the ps timescale (the EET rate according to the Förster mechanism) which is modulated by CO_2_ in ApcAB^WT^ (αβ)_3_. In the absence of CO_2_, we observed a long lifetime component significantly longer than could be fit with the period of data acquisition. The lifetime was significantly reduced in the presence of CO_2_ and could be fit to give a value of 2.9 ps (τ_1_ = 200 fs (60% of the amplitude), τ_2_ = 2.9 ps (40% of the amplitude)). Crucially, in ApcAB^K6A^ (αβ)_3,_ the stimulated emission component was too long to be fit by the data and was also not observed to be reduced by CO_2_ within the sensitivity of the experiment. We conclude that this stimulated emission component is due to weaker coupling Förster EET within ApcAB (αβ)_3_. The modulation of this component with CO_2_ is consistent with the distance variations predicted in the MD simulations. Further, the Förster EET rates measured here are on a ps timescale and therefore compatible with the values calculated from MD simulations. In conclusion, ultrafast transient absorption measurements show an enhanced rate of Förster EET in ApcAB^WT^ (αβ)_3_ in the presence of CO_2_. Consequently, we propose that the CO_2_ enhanced EET rate reduces the yield of non-radiative decay early in the allophycocyanin EET sequence. Thus, the excitation energy is more effectively transferred to the final emitting state, resulting in a higher fluorescence QY.

### Allophycocyanin A CO_2_-binding regulates EET in the cell

We next assessed whether CO_2_/HCO_3_^−^ enhanced EET in the context of the whole organism. QY measurements from the PBS in vivo are typically an order of magnitude lower than for the isolated recombinant proteins. This decreased QY is due to efficient EET from the PBS to the photosystem via the terminal pigment and subsequent photochemistry (charge separation and electron transfer) at the reaction centres. Therefore, the observed QY in vivo reflects energy lost from the PBS that is not transmitted to the photosystem. However, we reasoned that an increased observed QY would be reflective of increased EET to the photosystem due to reduced non-radiative decay at the initially excited chromophores, as previously discussed.

**Fig. 5:**
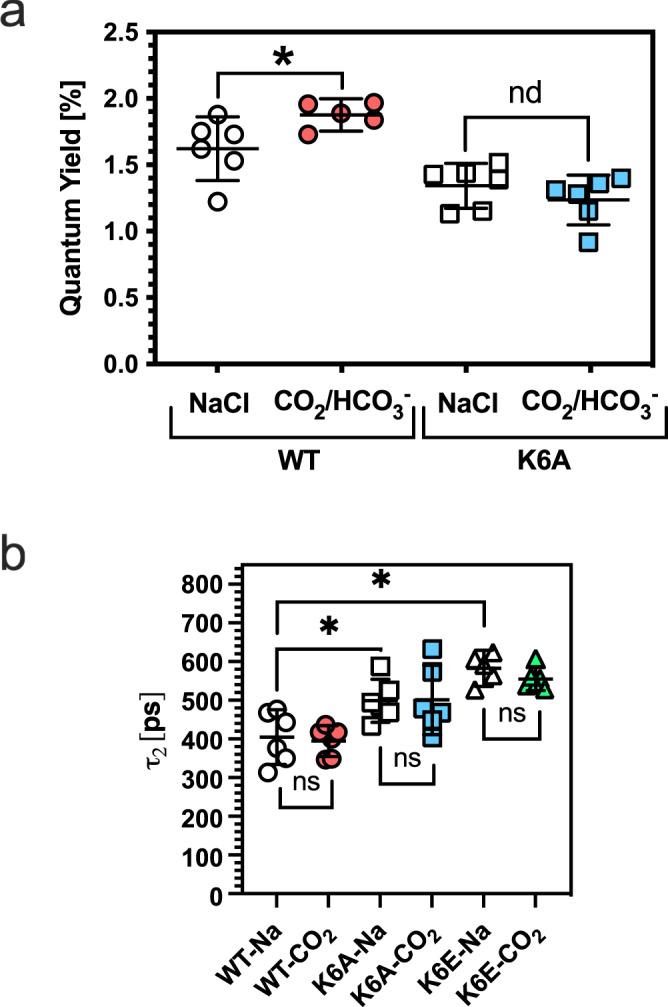
CO_2_ enhances ApcAB QY in vivo. **a** The measured fluorescence QY with 1 mM NaHCO_3_ (CO_2_/HCO_3_^−^) or NaCl for *Synechocystis* sp. PCC 6803 wild type or K6A cells (*q = 0.0058, one-way ANOVA with post hoc two-stage linear set-up procedure of Benjamini, Krieger and Yekutieli, *n* = 6 independent replicates for WT + NaCl, K6A + NaCl and K6A + CO_2_, *n* = 5 independent replicates for WT + CO_2_, *t* = 2.382, *df* = 19, nd no discovery, mean ± 95% *CI*). **b** The second fluorescent lifetime component (Table 1) with 1 mM NaHCO_3_ (-CO_2_) or NaCl (-Na) for *Synechocystis* sp. PCC 6803 wild type, K6A, or K6E cells (*p < 0.05, ns = not significant, two-way ANOVA of data from Table 1 with post hoc Tukey test, *n* = 6 independent replicates, mean ± 95% CI). Source data are provided as a Source Data file.

We used CRISPR-Cpf1 to generate *Synechocystis* sp. PCC 6803 with single amino acid mutations at K6 in ApcA. Absorption spectra were measured for wild type, K6A and K6E cells and were broadly similar (Supplementary Fig. 16). The relative peak heights for the PBS were similar in all strains (Supplementary Fig. 16 inset). The peak height for P680 for K6E cells was increased. As this absorption at the photosynthetic reaction centre is downstream of the effects studies at the PBS, this strain was still investigated further. We depleted *Synechocystis* sp. PCC 6803 wild type and K6A cells of CO_2_/HCO_3_^−^ under cool white light and measured the fluorescence QY when exciting chlorophyll *a* and PBS at 485 nm and measuring emission at 660 nm (Fig. 5a). Although 485 nm will also excite carotenoids, their low QY and efficient electronic energy transfer to chromophores makes it very unlikely they will impact on the observations. We observed a slight but significant enhancement of QY for wild type cells but not K6A cells when exposed to CO_2_/HCO_3_^−^. The observed effect is small, as expected, as energy transfer from the PBS to the photosystem is extremely efficient. However, both the small size of the effect and the indirect measure of EET limited the utility of this approach. We, therefore, measured whole-cell fluorescence lifetimes at 660 nm for wild type, K6A and K6E cells excited at 635 nm (Table 1; Fig. 5b). Two predominant fluorescent lifetimes on the ps timescale were observed. No difference in lifetime or fluorescence amplitude was observed between cells in the presence of NaCl or CO_2_/HCO_3_^−^ within the measurement error for the experiment. However, the τ_2_ fluorescence lifetime for both K6A and K6E cells was significantly longer than for wild type cells, indicating increased energy transfer to the emitting species (Fig. 4b). This observation is consistent with the increased basal fluorescent QY for ApcAB^K6A^, ApcAB^K6E^ (αβ)_3_ trimers compared to wild type (Fig. 2c) and decreased inter-chromophore distance (Fig. 3d). The 635 nm excitation wavelength can also excite phycocyanin. However, as the effect on the τ_2_ fluorescence lifetime is dependent on allophyocyanin genotype (Table 1), the impact of phycocyanin is likely minimal but cannot be ruled out.

**Table 1:** Fluorescent lifetimes for *Synechocystis* sp

	τ_1_ (ps)	RA (%)	τ_2_ (ps)	RA (%)	<τ> (ps)
WT + Na	164 ± 30	82	410 ± 74	18	252 ± 65
WT + Ci	154 ± 22	79	395 ± 38	21	251 ± 62
K6A + Na	163 ± 19	77	499 ± 53	23	324 ± 89
K6A + Ci	168 ± 28	80	501 ± 85	20	315 ± 100
K6E + Na	162 ± 13	76	583 ± 38	24	380 ± 29
K6E + Ci	156 ± 18	69	555 ± 29	31	403 ± 65

PCC 6803 wild type, K6A, or K6E cells in the presence of NaCl (+Na) or CO_2_/HCO_3_^−^ (+Ci). *RA* = Relative Amplitude. *n* = 6 independent replicates. Source data are provided as a Source Data file.

Together, the data support the hypothesis that carbamate formation at ApcA K6 breaks a salt bridge with D100 and enhances EET in whole cells.

## Discussion

The Calvin–Benson–Bassham (CBB) cycle rate depends on multiple factors. However, PCO_2_ is particularly important as it determines the relative rate of RuBisCO carboxylation compared to the competing oxygenation reaction. Following ribulose-1,5-biphosphate carboxylation, the CBB cycle requires ATP and NADPH, whose availability is controlled by light-dependent reactions. Therefore, although the light-dependent reactions might be independent of substrate availability, reduced PCO_2_ could represent a bottleneck for the ensuing light-independent reactions. It follows that an organism should optimise the light-dependent reaction rate to fit the changing environmental conditions that, under specific adverse scenarios, could lead to damage of the photosystems. Therefore, it seems paramount that CO_2_ can have a regulatory effect at the level of light-harvesting and/or light-dependent reactions for ‘sink to source’ regulation. The finding of a CO_2_-mediated PTM in the PBS might represent such a mechanism.

## Methods

### CO_2_ trapping

All CO_2_ trapping experiments were conducted in phosphate buffer (4 mL, 50 mM, pH 7.4). The solution was transferred to a potentiometric titrator (902 Titrando; Metrohm) and incubated at 25 °C with stirring. Next, a freshly made solution of triethyloxonium tetrafluoroborate (Et_3_OBF_4_; (280 mg, 1.47 mmol) in dH_2_O (1 mL)) was added stepwise with a constant pH maintained (pH 7.4) through the slow addition of 1 M NaOH solution via the automatic burette. The reaction mixture was stirred, and the pH was maintained for 1 h after the final Et_3_OBF_4_ addition to ensure that all TEO was hydrolysed. The reaction mixture was then dialysed against dH_2_O (1 L) overnight.

### Mass spectrometry

The post-dialysis reaction trapping sample supernatant was removed using vacuum centrifugation. The dried protein sample was resuspended in 8 M urea and reduced with dithiothreitol (DTT, 25 mM final concentration) at 37 °C for 1 h. The sample was then alkylated with iodoacetamide (40 mM final concentration) in the dark for 1 h. This sample was then centrifuged at 1000 g for 5 min and the soluble supernatant removed. The sample was diluted to 1 M urea and digested with trypsin gold (mass spectrometry grade, Promega) in a 1:25 (w/w) ratio overnight at 37 °C. The digested solution was then desalted on a C18 column and analysed by ESI-MS/MS on an LTQ Orbitrap XL mass spectrometer (Thermo) coupled to an Ultimate 3000 nano-HPLC instrument. Peptides eluted from the LC gradient were injected online to the mass spectrometer (lock mass enabled, mass range 400–1800 Da, resolution 60,000 at 400 Da, 10 MS/MS spectra per cycle, collision-induced dissociation (CID) at 35% normalised CE, rejection of singly charged ions). The post-run raw data files were converted into.mgf files using the freeware MSConvert provided by Proteowizard^29^. mgf files were analysed using PEAKS Studio 10.5 software^30^, including the variable modifications ethylation (28.03 at D or E), carboxyethylation (72.02 at K or protein *N*-terminal groups), oxidation (M), acetylation (N-terminal) and the fixed modification carbimidomethyl (C). These data were then refined using a false discovery rate of 1%, two unique peptides per protein and a PTM AScore of 50. Three carbamates PTMs were discovered (including on ApcA). The two additional carbamate PTMs will be reported elsewhere.

### NMR

Recombinant allophycocyanin alpha subunit wild type and K6A mutant protein were prepared in phosphate saline buffer, pH 7.4, 50 mM NaH^13^CO_3_ with 20–50% D_2_O. ^13^C-NMR experiments spectra were acquired with a Varian 600 MHz spectrometer equipped with an Agilent OneNMR Probe to deliver a maximum pulsed-field gradient strength of 62 G cm^−1^. A ^1^H spectrum was acquired to examine for small molecule impurities. Thirteen ^1^H experiments were recorded in 12 h, collecting 131072 complex points. The repetition time was 6.7 s, of which 1.7 s comprised the acquisition time. The excitation pulse angle was set to 45 degrees. The strong interfering H_2_O signal was eliminated using the Robust-5 pulse sequence^31^. Thirty-two ^13^C scans were collected, comprising 65 536 complex data points and a spectral width of 10 kHz. The repetition time was 6.3 s, of which 3.3 s comprised the acquisition time. The W5 inter-pulse delay was set to 240 µs. Rectangular 1 ms pulsed-field gradients were used in all cases with a strength of G1 = 28.3 G cm^−1^ (first pair) and G2 = 4.9 G cm^−1^ (second pair). The gradient stabilisation delay was 0.5 ms. The first pair of lock pre-focusing field gradients were separated from the first radio-frequency pulse by a 1.5 ms delay.

### Cyanobacterial genetic manipulation

*Synechocystis* sp. PCC 6803 was cultivated in a BG-11 liquid medium with a pH of 8.0 at 28 °C. Phycobilisome isolation was performed according to the method of ref. 32. The plasmid pSL2680 containing *cpf1*, the native *Francisella novicida* CRISPR array and *LacZ*, amplified from the pCrispomyces-2, served as the base plasmid for construction of slr2067 K6 editing plasmids^33^. pSL2680 expressing full-length pre-crRNAs was constructed by cloning annealed oligonucleotides (5′- AGA TAT TTC GTG ACG ATA CTC ATG G-3′ and 5′- AGA CCC ATG AGT ATC GTC ACG AAA T-3′) into the *Aar*I sites to yield pSL2680apcAgRNA. PCR was used to synthesise the homology regions which were cloned into the KpnI site on the plasmids containing the matching crRNA. The point mutation homology region was constructed in two pieces using oligonucleotides 5′-CAT TTT TTT GTC TAG CTT TAA TGC GGT AGT TGG TAC CCT TTA ATA AGC TTG GGT ACA CAG AC-3′/ 5′-GCT TCT GCA TCA GCA TTC ACG ATC GAT GCC GTG ACG ATA CTC ATG G-3′ and 5′- GCC CGG ATT ACA GAT CCT CTA GAG TCG ACG GTA CCG GCT CAC CTG TGA TGC CAT TG-3′/5′-CCA TGA GTA TCG TCA CGG CAT CGA TCG TGA ATG CTG ATG CAG AAG C-3′ for the K6A mutation and 5′-CAT TTT TTT GTC TAG CTT TAA TGC GGT AGT TGG TAC CCT TTA ATA AGC TTG GGT ACA CAG AC-3′/ 5′-GCT TCT GCA TCA GCA TTC ACG ATC GAT TCC GTG ACG ATA CTC ATG G-3′ and 5′- GCC CGG ATT ACA GAT CCT CTA GAG TCG ACG GTA CCG GCT CAC CTG TGA TGC CAT TG-3′/5′-CC ATG AGT ATC GTC ACG GAA TCG ATC GTG AAT GCT GAT GCA GAA GC-3′ for the K6E mutation. The homology templates were assembled, linearised with *Kpn*I, and PCR was used to generate pSL2680-K6AapcA and pSL2680-K6EapcA. *E. coli* with cargo plasmid either pSL2680-K6AapcA or pSL2680-K6EapcA and *E. coli* with conjugal plasmid pRL443 were grown overnight at 37 °C. 10 mL of each culture was pelleted and resuspended in 10 mL LB media without antibiotics. Cargo and conjugal cultures were mixed and 200 µl combined with 100 µl of *Synechocystis* culture. The mixture was incubated for 5 h at 30 °C before plating on BG11 agar plates without antibiotics. Plates were incubated for 2 days at 30 °C. 15 μg mL^−1^ kanamycin was added, and plates were incubated at 30 °C with light for 10 days until colonies appeared. At least four rounds of streak purification were performed to ensure complete segregation. Colonies were cured of plasmids on BG11 agar without antibiotics. Two mutant strains were produced for each point mutation.

### Cyanobacterial manipulation for spectroscopy

*Synechocystis* sp. PCC 6803 cells were grown at 30 °C with constant shaking in BG-11 pH 8 medium under continuous cool-white fluorescent light at 30 μmol photons m^−2^ s^−1^. Cells were harvested at the late exponential phase by centrifugation, washed in degassed BG11 media, and resuspended with fresh degassed BG11 to an optical density of ~1.0 at 730 nm. Cells were incubated in a Clark type O_2_ electrode chamber under light at 25 °C until no further O_2_ was evolved, indicating CO_2_ depletion.

### Recombinant protein production

The ApcAB protein of *Synechococcus* sp. PCC 7002 is 86.3 % identical and 93.8 % similar to the ApcAB protein of *Synechococcus* sp. PCC 6803 (Supplementary Fig. 1b). The ApcAB protein from *Synechococcus* sp. PCC 7002 can be produced as a recombinant in *Escherichia coli* as a recombinant protein and was, therefore, used for in vitro biochemistry. Expression plasmid carrying *Synechococcus* sp. PCC 7002 ApcAB were cotransformed into *E. coli* BL21(DE3) cells with plasmids for chromophorylation. A 50-ml starter culture was added to 1 L of LB medium with the appropriate combination of antibiotics and shaken at 37 °C for 4 h until the optical density at 600 nm (OD600) was 0.6. Recombinant protein production was induced by addition of 0.5 mM isopropyl-γ-D-thiogalactoside (IPTG). Cells were incubated with shaking at 30 °C for another 4 h before they were harvested by centrifugation at 10,000 × *g*. Cells containing recombinant protein were thawed and resuspended in buffer O (50 mM Tris-HCl, 150 mM NaCl; pH 8.0) at 1 g (wet weight) cells/2.5 ml and then were lysed by French Press. The lysed cell suspension was centrifuged at 13,000 × *g*, and the recombinant protein purified using a nickel-nitrilotriacetic acid column^34^.

Plasmid construction for production of recombinant *Synechococcus* sp. PCC 7002 ApcAB mutant plasmid was performed using standard molecular techniques and mutagenic primers 5′-ATG AGT ATT GTC ACG GCA TCC ATC GTG AAT GCC GAC-3′ and 5′-GTC GGC ATT CAC GAT GGA TGC CGT GAC AAT ACT CAT-3′ for K6A and 5′-GTC ACG GAA TCC ATC GTG AAT GCC GAC GCT G-3′ and 5′-GAT GGA TTC CGT GAC AAT ACT CAT GGT GAA GGG ATG-3′ for K6E.

### Molecular Dynamics simulations

Comparing the structures of (αβ)_3_ allophycocyanin trimers from *Thermosynechococcus vulcanus* (PDB: 3DBJ [https://www.rcsb.org/structure/3DBJ])^25^, *Synechococcus* sp. PCC 7002 (PDB: 7EXT [https://www.rcsb.org/structure/7EXT])^35^ and *Synechocystis* PCC 6803 (PDB: 4PO5 [https://www.rcsb.org/structure/4PO5])^36^, shows that they are very similar (Supplementary Fig. 13). Accordingly, structural superpositions provided low RMSD values between the (αβ)_3_ trimers; 0.78 Å RMSD between trimers from 3DBJ and 4PO5, 1.56 Å RMSD between trimers from 3DBJ and 7EXT, and 1.82 Å RMSD between trimers from 4PO5 and 7EXT. All-atom molecular dynamics simulations of *Thermosynechococcus vulcanus* (PDB: 3DBJ [https://www.rcsb.org/structure/3DBJ]) ApcAB (αβ)_3_ trimer in its apo and CO2-bound form were carried out using the AMBER18 molecular modelling software^37^. The topology and the initial coordinate files were generated using the LEaP module of AmberTools18. The systems simulated features no histidine, all other titratable groups were assigned to their default protonation state at 7.4 pH. The ff14SB^38^ version of the all-atom Amber force field was used to represent the protein systems. The parameters for chromophore and non-standard amino acid residues (N-methyl asparagine and CO_2_-bound lysine) were taken from the GAFF2 forcefield^39^, and the missing ones were generated with Antechamber^40^ (see Supplementary Data file). Each system was solvated with an explicit TIP3P water model and neutralised with the required number of randomly placed K^+^/Cl^−^ ions. The ion count was adjusted to account for an osmolarity of 0.15 M.

The systems were independently subjected to 2-step energy minimisation with a combination of steepest descent and conjugate gradient methods using the pmemd programme implemented in AMBER to relax internal constraints by gradually releasing positional restraints. The first minimisation step had positional restraints on protein backbone atoms (Cα, C, N) and the chromophore with a restraint force constant of 10.0 kcal/mol/Å^2^, which were then removed in the second minimisation step. Following this, the systems were gradually heated to 300 K under constant volume (NVT) for 500 ps with positional restraints (restraint force constant of 5.0 kcal/mol/Å^2^) on the protein Cα atoms and the entire ligand. They were then density equilibrated for 5 ns under constant pressure (NPT) to bring the atmospheric pressure of the system to 1 bar. Microsecond long production run was performed for each system under NPT conditions. Langevin thermostat (collision frequency of 1 ps^−1^) was used to maintain a constant temperature and Berendsen barostat for pressure scaling. An integration time step of 2 fs was used, with all bonds to hydrogen atoms constrained with the SHAKE algorithm. The particle-mesh Ewald (PME) algorithm was used to evaluate long-range electrostatic forces with a non-bonded cut-off of 9 Å. Coordinates were saved every 10 ps. MD trajectories were analysed using the cpptraj module of AmberTools18 and VMD1.9.2^41^.

### Steady-state absorption and fluorescence spectroscopy

Absorption spectra and fluorescence spectra were obtained using a Synergy H4 Plate Reader and/or a Cary 5000 UV-Vis-NIR. Steady-state fluorescence was measured using a Jobin Yvon Horiba Fluorlog 3. Photoluminescence quantum yield measurements on solutions were carried out according to the absolute method, using an integrating sphere. The optical density of recombinant proteins was adjusted to 0.1 at their fluorescence maximum. For in vitro PLQY measurements using recombinant protein, measurements were made before and after the addition of 20 mM CO_2_/HCO_3_^−^ or NaCl to calculate the impact of the addition. Data is presented at the ratio of PLQYs with CO_2_/HCO_3_^−^ or NaCl.

### Circular dichroism

Spectra were collected in high transparency quartz cuvettes with a 0.1 cm path length using a Jasco J-1500 spectropolarimeter equipped with a mini-circulation bath and Peltier stage. Spectra for secondary structure determination were measured from 190–260 nm in PBS, pH 7.4, at a protein concentration of 12.6 μM.

### Time-correlated single photon counting

Time-correlated single-photon counting (TCSPC) was carried out using a DeltaFlex. The data were subsequently fitted to a sum of exponentials;

$$F(t)=\mathop{\sum}\limits_{i}{A}_{i}\exp (-{k}_{i}t)$$

by deconvolution with the instrument response function (IRF). The IRF was obtained through light scattering from Ludox particles dispersed in solution. The FWHM was ~200 ps, which afforded a temporal time-resolution of ≥100 ps.

### Ultrafast transient absorption spectroscopy

Ultrafast transient absorption spectroscopy was performed using a custom-built transient absorption (TA) set-up^42^. All laser pulses used were derived from a Yb:KGW laser system (Carbide, Light Conversion), providing pulses with 1028 nm, 54 µJ, 250 fs at 60 kHz repetition rate. Approximately 4 µJ pulse^−1^ was used to generate white light (WL) generation in a 5 mm-thick sapphire window, which served as the probe (range ~550–850 nm). The remaining ~50 µJ pulse^−1^ was used to generate 514 nm light by second-harmonic generation in a BBO crystal, which served as the pump. The polarization of the probe was at the magic angle (54.7°) relative to the pump. The pump pulse train was chopped at 30 kHz, attenuated to deliver ~0.8 µJ pulse^−1^ at the sample, and delayed relative to the pump using a motorised translation stage. Both pump and probe pulses were independently focussed onto the sample using curved mirrors. The transmitted WL was recollimated using a lens. A polariser was used to minimise the scattering of the pump by the sample. The WL was passed through a slit to select the central portion of the beam and dispersed via a prism onto a line-scan camera (LightWise Allegro, Imaging Solutions Group). The sample was contained in a standard cuvette of 1 mm path length, which was translated to reduce sample degradation. TA experiments on a given sample typically took 10 min to complete, which avoided overall sample degradation. The concentration of samples was 5 mg ml^−1^.

### Statistical analysis

All error bars represent a 95% confidence interval. All statistics and graphical analyses were performed using GraphPad Prism 8 (GraphPad Software, inc.). All data points represent independent experiments.

### Reporting summary

Further information on research design is available in the Nature Research Reporting Summary linked to this article.

## Supplementary information

Supplementary Information

Reporting Summary

## Data Availability

The authors declare that the data supporting the findings of this study are available within the article, the supplementary information file, and Source Data file. The structural data used in this study are available in the Protein Databank database under accession codes 3DBJ, 7EXT and 4PO5. Source data are provided with this paper.

## Source data

Source Data
