# Supplementary material for: Allophycocyanin A is a carbon dioxide receptor in the cyanobacterial phycobilisome
Source: Nat Commun. 2022 Sep 8;13:5289. doi: 10.1038/s41467-022-32925-6 (PMC9458709; doi:10.1038/s41467-022-32925-6)
Supplement: Supplementary file 1 — Supplementary Information [file 41467_2022_32925_MOESM1_ESM.pdf]

Supplementary information.

Allophycocyanin A is a carbon dioxide receptor in the cyanobacterial phycobilisome.

Alejandra Guillen-Garcia[1#], Savannah E.R. Gibson[1#], Caleb J.C. Jordan[2], Venkata K. Ramaswamy[3], Victoria L. Linthwaite[1], Elizabeth H.C. Bromley[3], Adrian P. Brown[1], David R.W. Hodgson[3,4], Tim R. Blower[1,4], Jan R.R. Verlet[2], Matteo Degiacomi[3,4], Lars-Olof Pålsson[2,4], Martin J. Cann[1,4*].

[1]Department of Biosciences, [2]Department of Chemistry [3]Department of Physics, [4]Biophysical Sciences Institute, Durham University, South Road, Durham DH1 3LE, United Kingdom.

[#] These authors contributed equally to this work

* Address correspondence to: Martin J Cann, Department of Biosciences, Durham University, South Road, Durham, DH1 3LE, United Kingdom. Phone: +44 (191) 3343985. E-mail: m.j.cann@durham.ac.uk.

E-mail: m.j.cann@durham.ac.uk

Keywords
Carbon dioxide; carbamate; cyanobacteria; phycobilisome.

A.

```
MSIVTKSIVNADAEARYLSPGELDRIKAFVTGGAARLRIAETLTGSRETIV
KQAGDRLFQKRPDIVSPGGNAYGEEMTATCLRDMDYYLRLVTYGVVSGDVT
PIEEIGLVGVREMYRSLGTPIEAVAQSVREMKEVASGLMSSDDAAEASAYF
DFVIGKMS
```

(In the sequence above, the K at position 6 is printed in bold and underlined.)

B.

```
Synechocystis sp. PCC6803       1   MSIVTKSIVNADAEARYLSPGELDRIKAFVTGGAARLRIAETLTGSRETI     50
                                    |||||||||||||||||||||||||||||||.|.:|||||||||||||.|
Synechococcus sp. PCC7002       1   MSIVTKSIVNADAEARYLSPGELDRIKAFVTSGESRLRIAETLTGSRERI     50

Synechocystis sp. PCC6803       51  VKQAGDRLFQKRPDIVSPGGNAYGEEMTATCLRDMDYYLRLVTYGVVSGD    100
                                    :|.|||.|||||||:||||||||||||||||||||||||||:|||||:||
Synechococcus sp. PCC7002       51  IKSAGDALFQKRPDVVSPGGNAYGEEMTATCLRDMDYYLRLITYGVVAGD    100

Synechocystis sp. PCC6803       101 VTPIEEIGLVGVREMYRSLGTPIEAVAQSVREMKEVASGLMSSDDAAEAS    150
                                    ||||||||||||||||:|||||::||||:|||||.||:|:||.||||||.
Synechococcus sp. PCC7002       101 VTPIEEIGLVGVREMYKSLGTPVDAVAQAVREMKAVATGMMSGDDAAEAG    150

Synechocystis sp. PCC6803       151 AYFDFVIGKMS    161
                                    ||||:|||.|.
Synechococcus sp. PCC7002       151 AYFDYVIGAME    161
```

Supplementary Figure 1. (A) The Slr2067 open reading frame (ApcA) of *Synechocystis* sp. PCC 6803. The carbamylated lysine is underlined in bold. **(B)** Protein sequence alignment calculated using the Needleman-Wunsch algorithm for the ApcA open reading frames of *Synechocystis* sp. PCC 6803 and *Synechococcus* sp. PCC 7002. Number indicates amino acid number, . (period) indicates conservation between groups of strongly similar properties (>0.5 in the Gonnet PAM 250 matrix), : (colon) indicates conservation between groups of weakly similar properties (=< 0.5 in the Gonnet PAM 250 matrix).

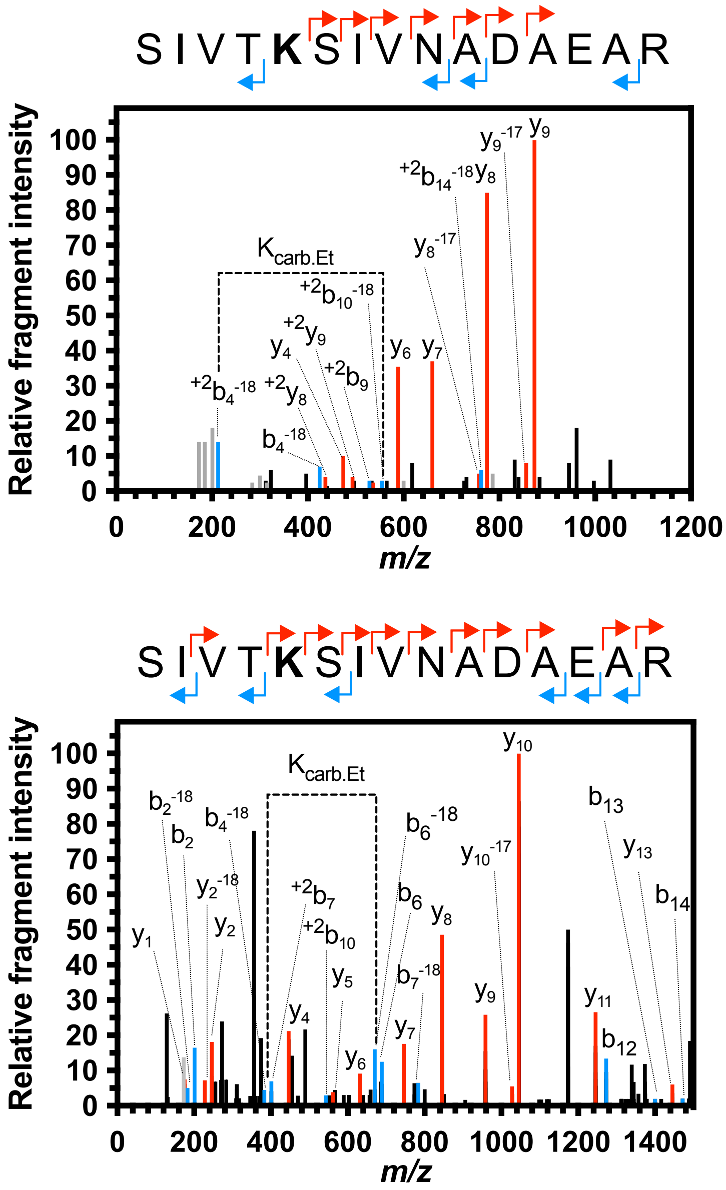

Supplementary Figure 2. CO_2 binds ApcA. Demonstration of CO_2-binding sites on ApcA by MS/MS. Plots of relative fragment intensity versus mass/charge ratio (m/z) for fragmentation data from MS/MS identifying ethyl-trapped carbamate on whole *Synechocystis* sp. PCC 6803 in the presence of $^{12}CO_2$. Peptide sequences indicate predominant +1y (red) +1b (blue) ions identified by MS/MS shown in the plot. The modified residue is indicated in bold. $K_{carb.Et}$ indicates the molecular weight difference between ions diagnostic of the modified Lys. Other observed ions, not labelled on the figure panels for clarity, are shown in grey and identified in Supplementary Table 2. The two

panels represent independent experiments on different *Synechocystis* sp. PCC 6803 preparations. Source data are provided as a Source Data file.

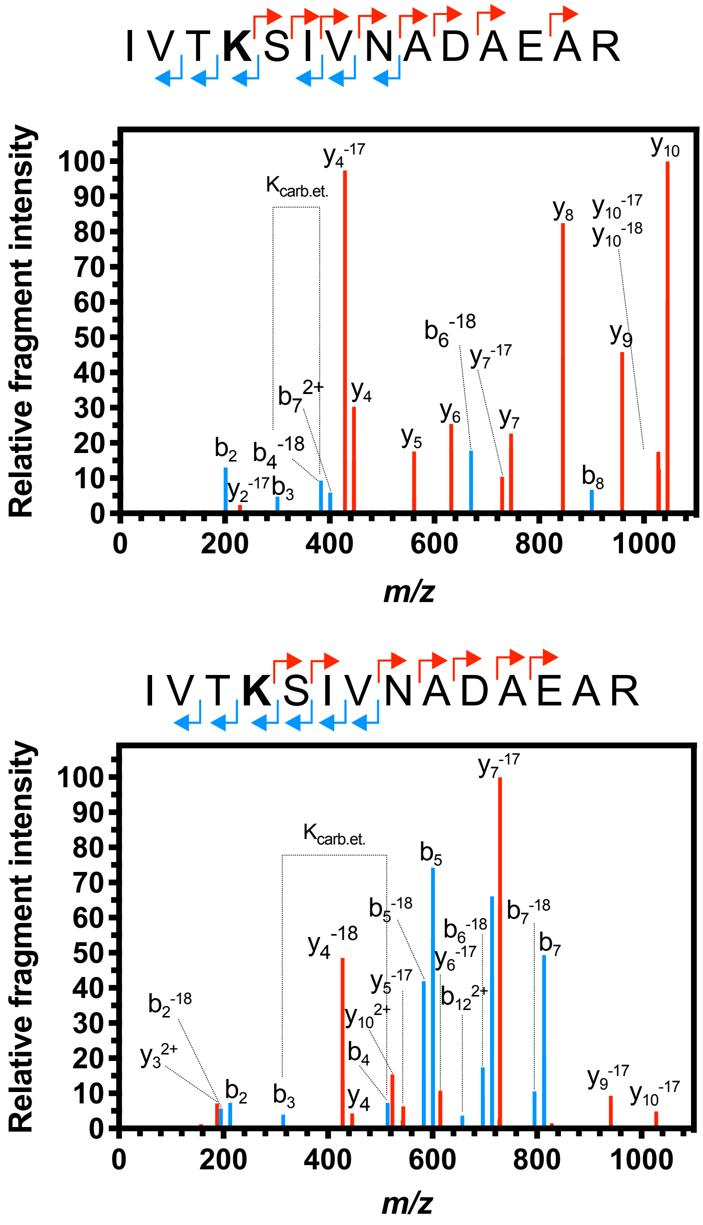

Supplementary Figure 3. CO_2 binds ApcA. Demonstration of CO_2-binding sites on ApcA by MS/MS. Plots of relative fragment intensity versus mass/charge ratio (m/z) for fragmentation data from MS/MS identifying ethyl-trapped carbamate on recombinant *Synechococcus* sp. PCC 7002 $ApcAB^{WT}$ $(\alpha\beta)_3$ trimers in the presence of $^{12}CO_2$. Peptide sequences indicate predominant +1y (red) +1b (blue) ions identified by MS/MS shown in the plot. The modified residue is indicated in bold. $K_{carb.Et}$ indicates the molecular weight difference between ions diagnostic of the modified Lys. The two panels represent independent experiments on different protein preparations. Source data are provided as a Source Data file.

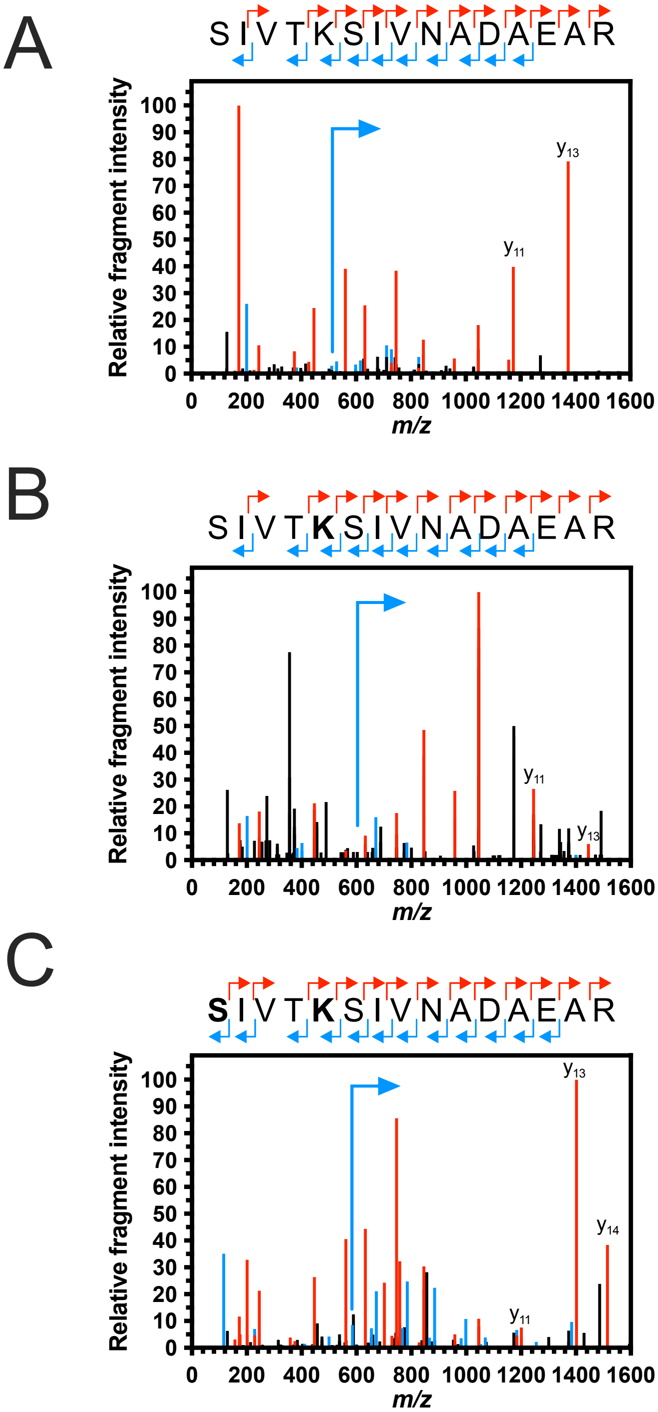

Supplementary Figure 4. CO_2 binds ApcA. Plots of relative fragment intensity versus mass/charge ratio (m/z) for fragmentation data from MS/MS identifying synthetic untrapped (**A**), K6 carboxyethyl modified (**B**) or S2K6 ethyl modified (**C**) SIVTKSIVNADAEAR peptide. Peptide sequences indicate +1y (red) +1b (blue) ions identified by MS/MS shown in the plot. The modified residues are indicated in bold. The y ions that cross K6 are labelled on the plots. The b ions to the right of the blue arrow

indicated on the plots cross K6. Identified ions are provided in Tables S4-6. Source data are provided as a Source Data file.

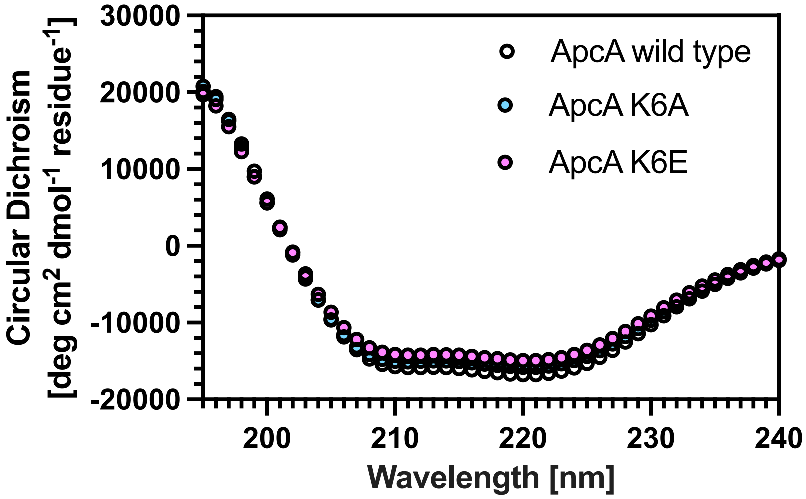

Supplementary Figure 5. Circular dichroism spectra for *Synechocystis* sp. PCC 6803 ApcA wild type, K6A, and K6E mutant proteins. Source data are provided as a Source Data file.

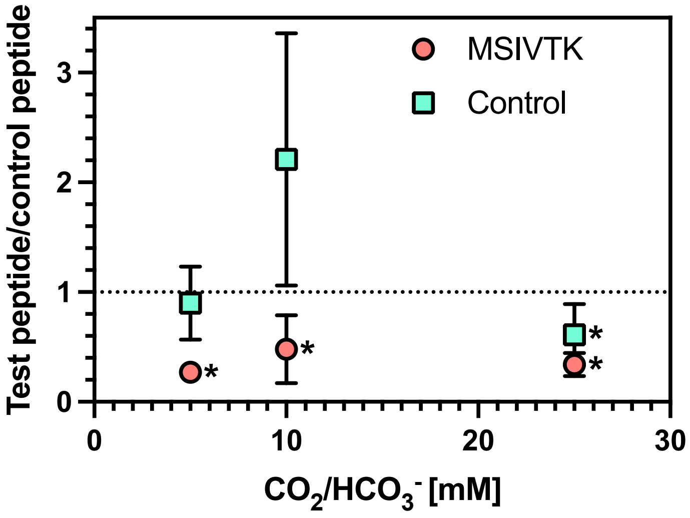

Supplementary Figure 6. Plot of the ratio of the peak area for a test peptide compared to a control peptide at varying CO_2/HCO_3^- normalized to the absence of CO_2/HCO_3^-. Therefore, *y*-axis value of 1 demonstrates that the incidence of this peptide is not different at *x* mM CO_2/HCO_3^- when compared to the absence of CO_2/HCO_3^-. A *y*-axis value <1 demonstrates that the incidence of this peptide is lower at *x* mM CO_2/HCO_3^- when compared to the absence of CO_2/HCO_3^-. MSIVTK is the ApcA peptide where K6 is carbamylated. The control peptides examined are YLSPGELDR, SIVNADAEAR, DMDYYLR, and TGGQQMGR. The data for the MSIVTK (four comparisons) and the control peptides (twelve pairwise comparisons) are aggregated into a single data point (*$p<0.05$, one sample t-test for theoretical mean = 1, $t>3.357$, $df>3$, ± S.D.). p values are Control 5 mM CO_2/HCO_3^- $p = 0.3208$, Control 10 mM CO_2/HCO_3 $p = 0.0038$ (note mean>1), Control 25 mM CO_2/HCO_3 $p = 0.0005$, MSIVTK 5 mM CO_2/HCO_3 $p = 0.0002$, MSIVTK 10 mM CO_2/HCO_3 $p = 0.0438$, MSIVTK 25 mM CO_2/HCO_3 $p = 0.0011$. Source data are provided as a Source Data file.

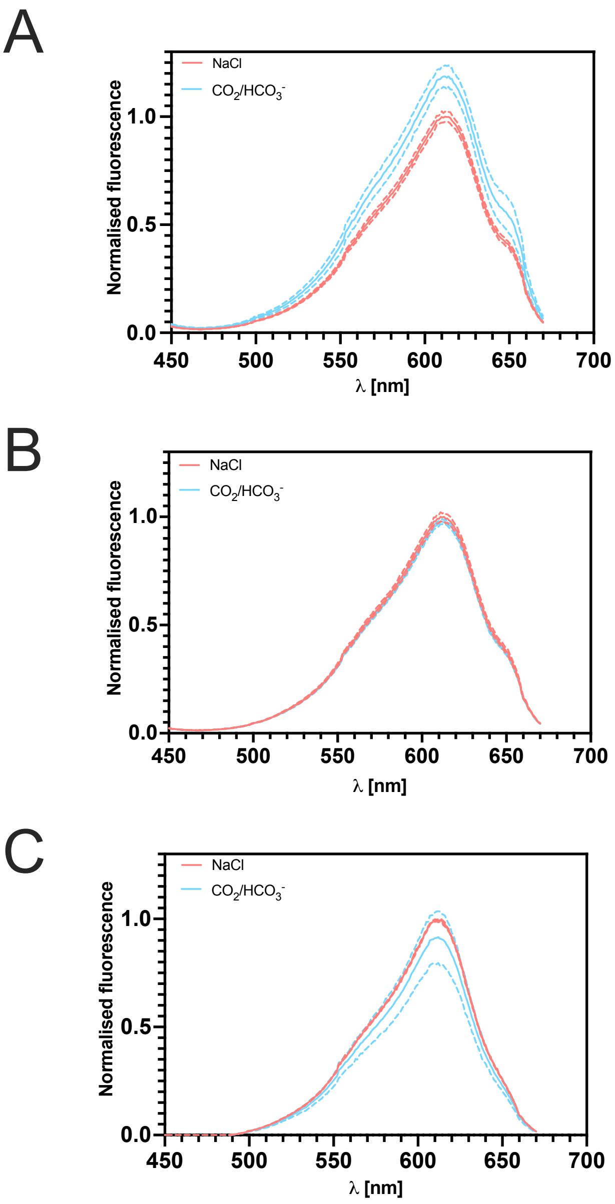

Supplementary Figure 7. Excitation spectra for recombinant wild type (**A**), K6A mutant (**B**) and K6E (**C**) ApcAB $(\alpha\beta)_3$ trimers. Graphs are plots of fluorescence normalized to the peak excitation wavelength in the presence of NaCl against excitation wavelength. Additives were 20 mM at pH 7.4. Dashed lines represent the standard deviation. Emission was monitored at 680 nm. Source data are provided as a Source Data file.

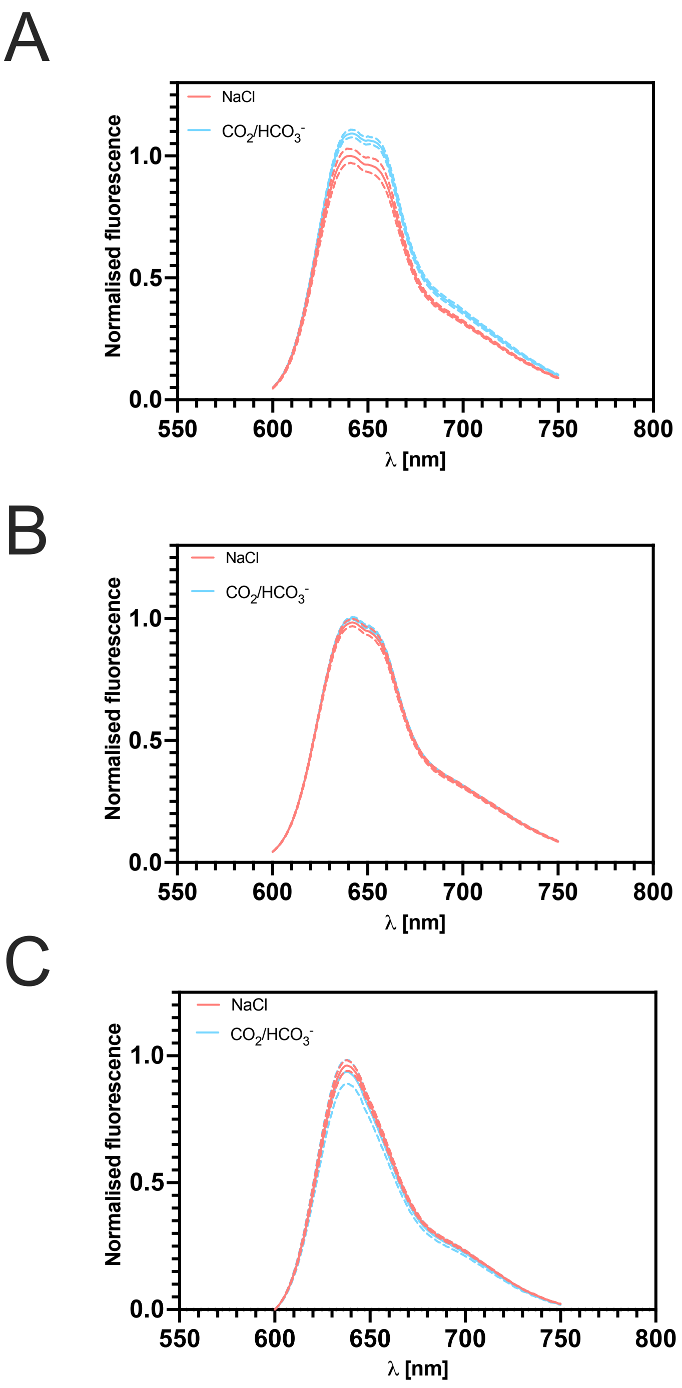

Supplementary Figure 8. Emission spectra for recombinant wild type (**A**), K6A mutant (**B**) and K6E mutant (**C**) ApcAB $(\alpha\beta)_3$ trimers. Graphs are plots of fluorescence normalized to the peak emission wavelength in the presence of NaCl against emission wavelength. Additives were 20 mM

at pH 7.4. Dashed lines represent the standard deviation. Source data are provided as a Source Data file.

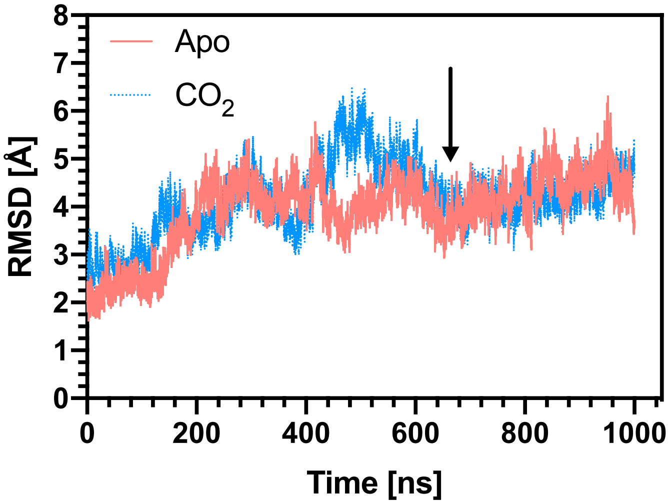

Supplementary Figure 9. Molecular dynamics simulation of ApcAB $(\alpha\beta)_3$ trimer in the apo and CO_2-bound form. Plot of protein backbone RMSD against simulation time. The simulation has stabilized after 600 ns (arrowhead). Source data are provided as a Source Data file.

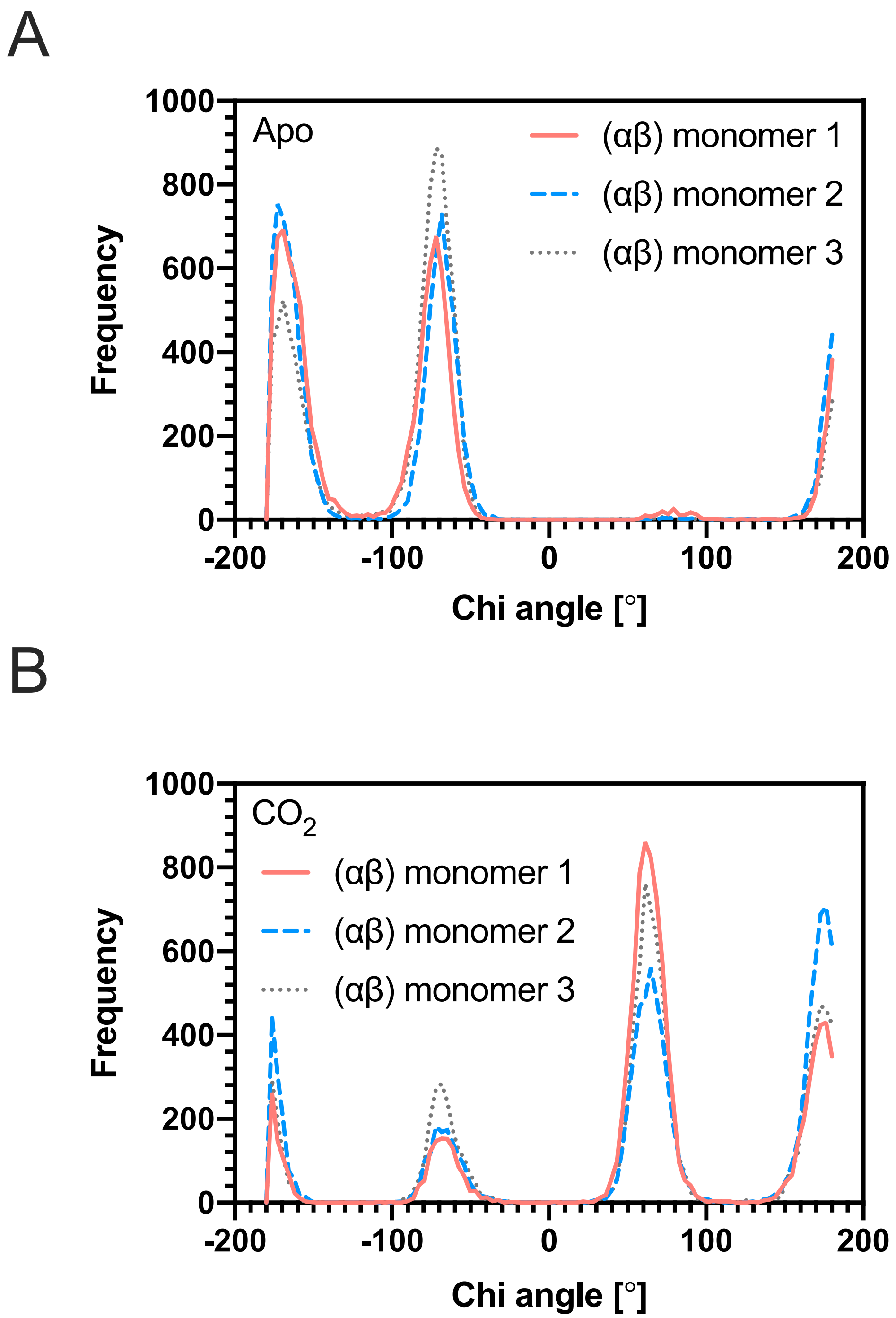

Supplementary Figure 10. Rotamer states for the ApcA lysine in the ApcAB (αβ)3 trimer in the apo and CO_2-bound form. Plot of angle frequency against the Chi angle for the CO_2-binding lysine of ApcA in each ApcAB (αβ) monomer in the apo (**A**) or CO_2-bound (**B**) form. Source data are provided as a Source Data file.

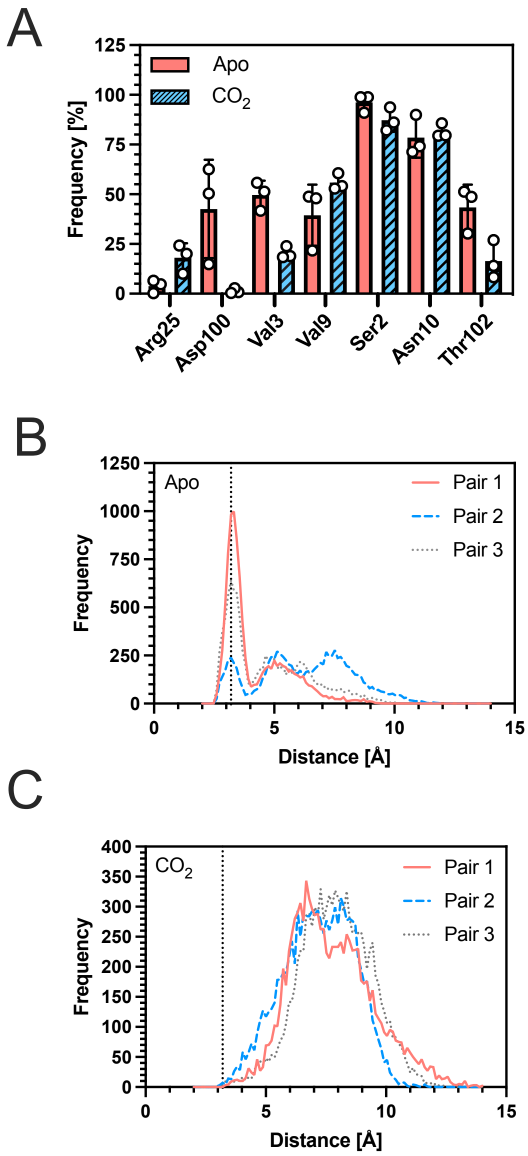

Supplementary Figure 11. ApcA forms a salt bridge between Lys6 and Asp100 in ApcAB $(\alpha\beta)_3$ trimers in the apo form. **A.** Plot of frequency of contact for Lys6 with neighboring residues in the apo or CO_2-bound state (± S.D., *n* = 3). **B-C.** Plot of frequency of observation against distance between the Lys6 and Asp100 side chains in the apo (**B**) or CO_2-bound (**C**) form. The dotted line at 3.2 Å indicates the distance for the hypothesized salt bridge. Source data are provided as a Source Data file.

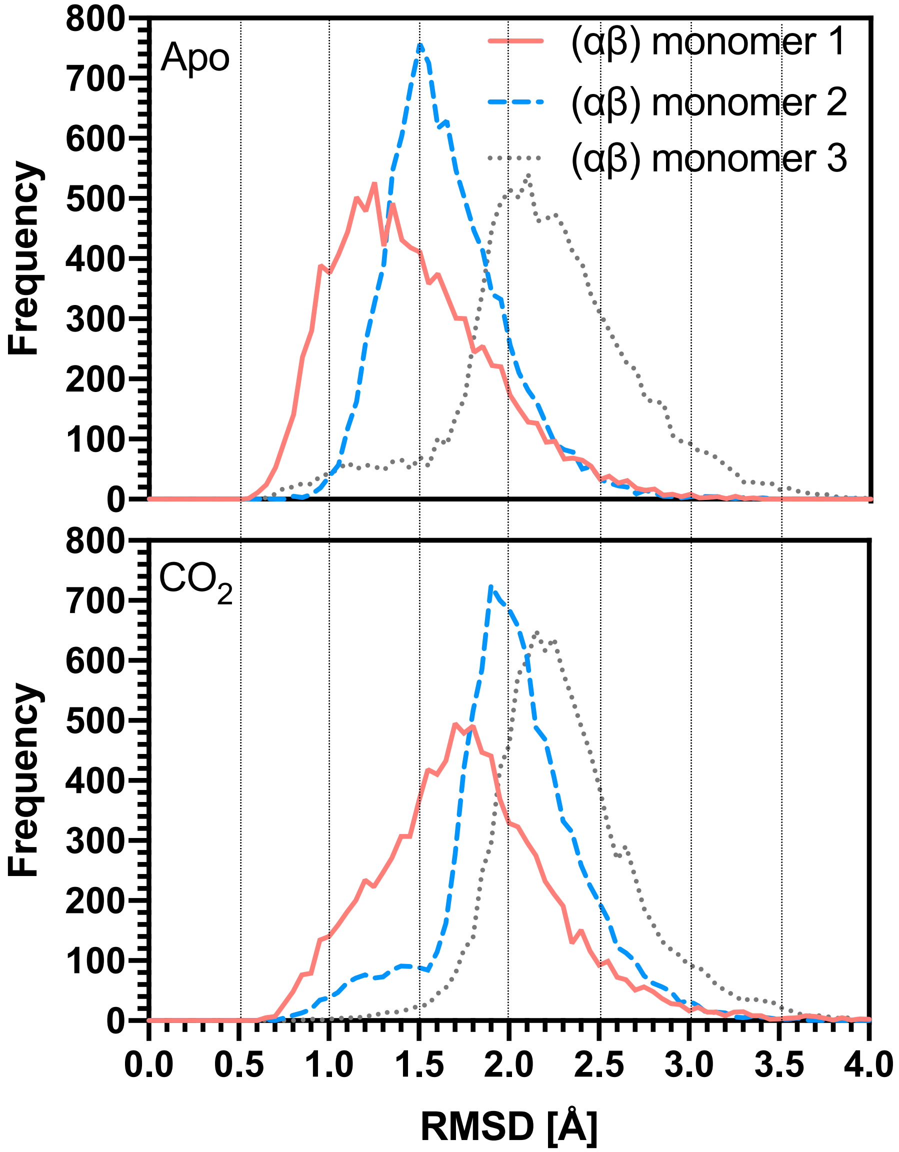

Supplementary Figure 12. CO_2 enhances the dynamics of an ApcAB ($\alpha\beta$) monomer α-helix. Plot of frequency of observation against the peptide backbone RMSD for the α-helix from ApcA amino acids 100-119 in the apo (top) and CO_2-bound (bottom) states for each of the three ApcAB ($\alpha\beta$) monomers. Two of the three α-helices show an increased backbone RMSD with CO_2. Source data are provided as a Source Data file.

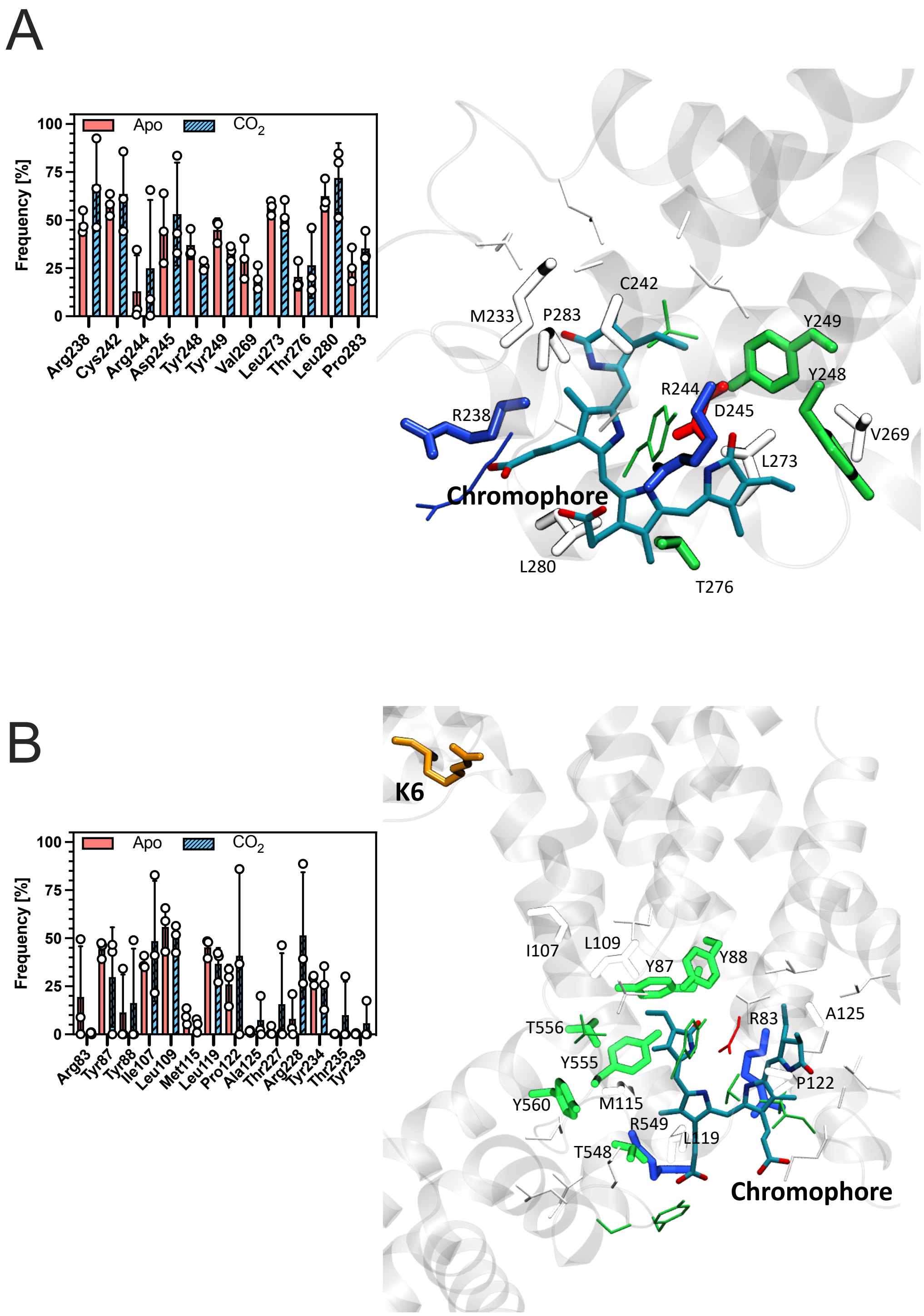

Supplementary Figure 13. CO_2 binding alters chromophore contacts. Left hand side of panels-Plots of contact frequency for chromophore-binding pocket residues for the monomer (**A**) and interface (**B**) pockets in the apo and CO_2-bound states averaged over the three ApcAB (αβ) monomers **(**± S.D., *n* = 3). Right hand side of panels-Cartoon of the chromophore-binding pockets for the monomer (**A**) and interface (**B**) pockets. All residues forming the chromophore-binding

pocket are shown in licorice (with different thickness) and colored by their residue type (acidic in red, basic in blue, polar in green and hydrophobic in white). Thicker lines show a difference in the contact frequency with chromophore between the ApcAB ($\alpha\beta$) dimer in the apo and CO_2-bound state. The chromophore is shown in cyan. Source data are provided as a Source Data file.

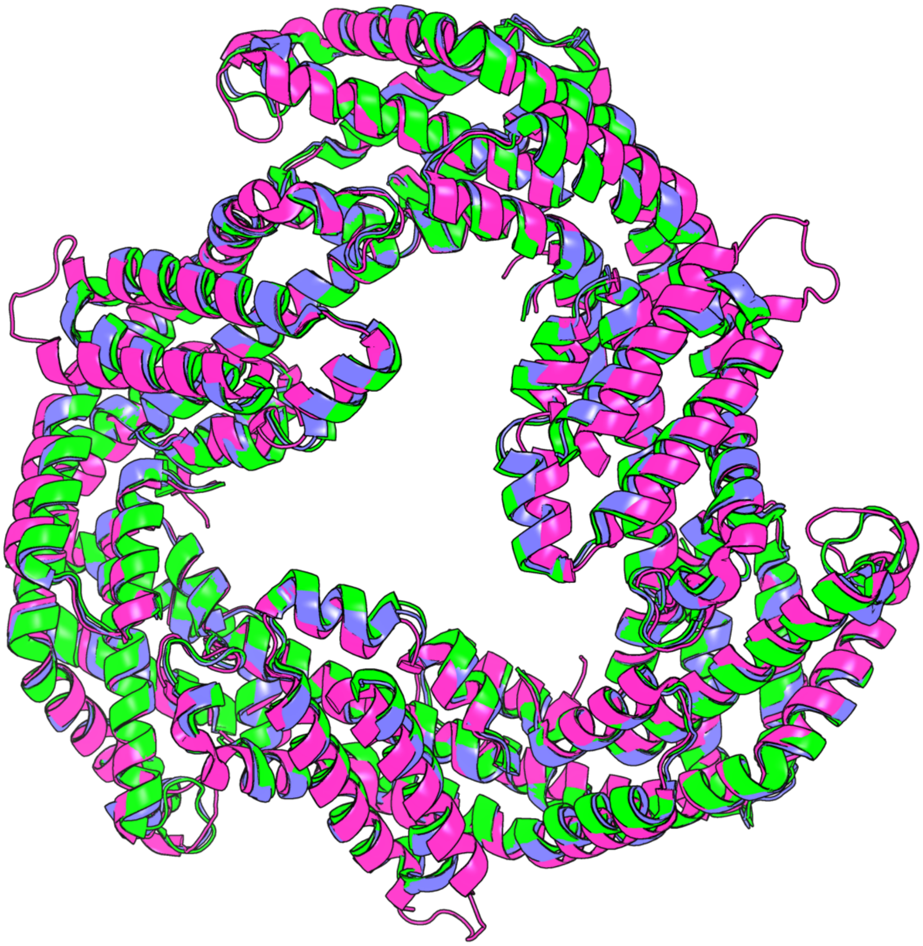

Supplementary Figure 14. Allophycocyanin (αβ)$_3$ trimers are very similar in structure. Superposed trimers are shown for *Thermosynechococcus vulcanus* (PDB: 3DBJ, blue [https://www.rcsb.org/structure/3DBJ]), *Synechococcus* sp. PCC 7002 (PDB: 7EXT, magenta [https://www.rcsb.org/structure/7EXT]) and *Synechocystis* PCC 6803 (PDB: 4PO5, green [https://www.rcsb.org/structure/4PO5]).

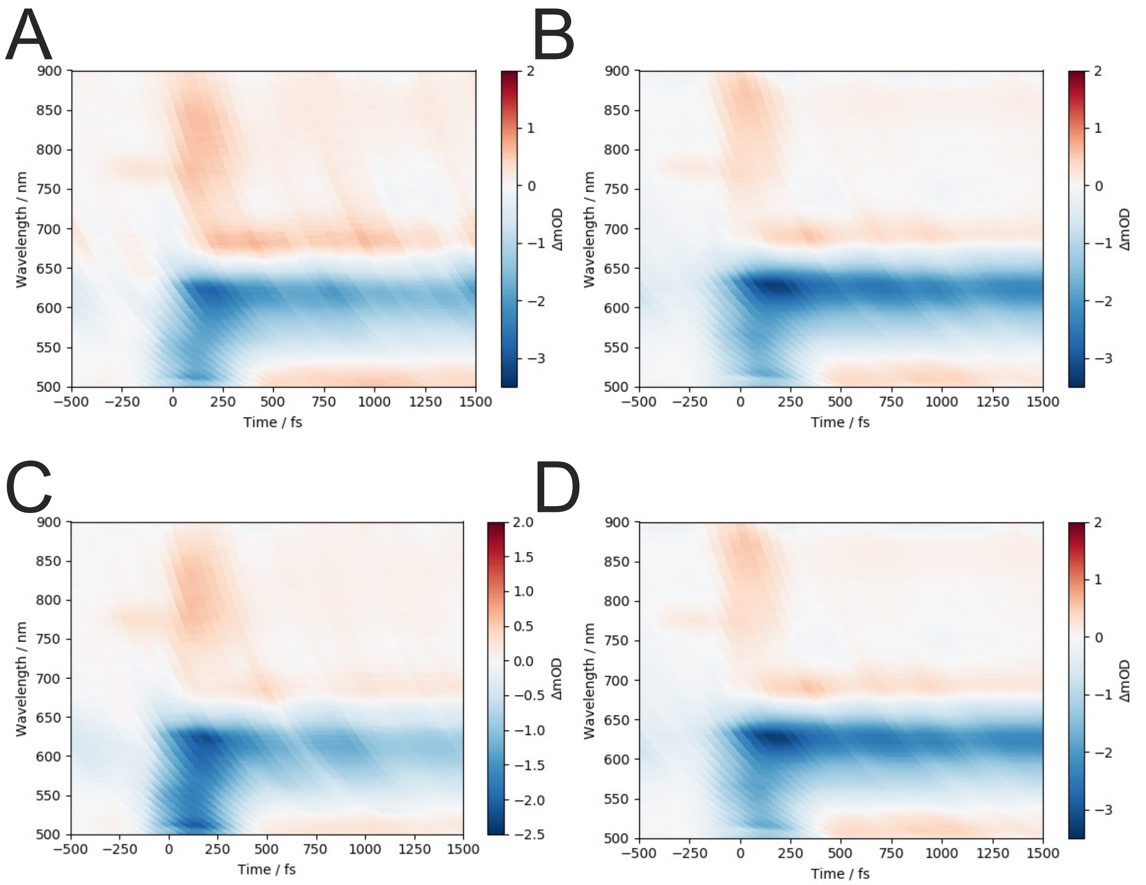

Supplementary Figure 15. Two-dimensional transient absorption maps from ultrafast transient absorption spectroscopy experiments for ApcAB^{WT} $(\alpha\beta)_3$ (**A**, **B**) and ApcAB^{K6A} $(\alpha\beta)_3$ (**C**, **D**) in the presence of NaCl (**B**, **D**) or CO_2/HCO_3^- (**A**, **C**). Maps are a plot of wavelength against time with the heat map representing change in optical density x10^{-3} (ΔmOD).

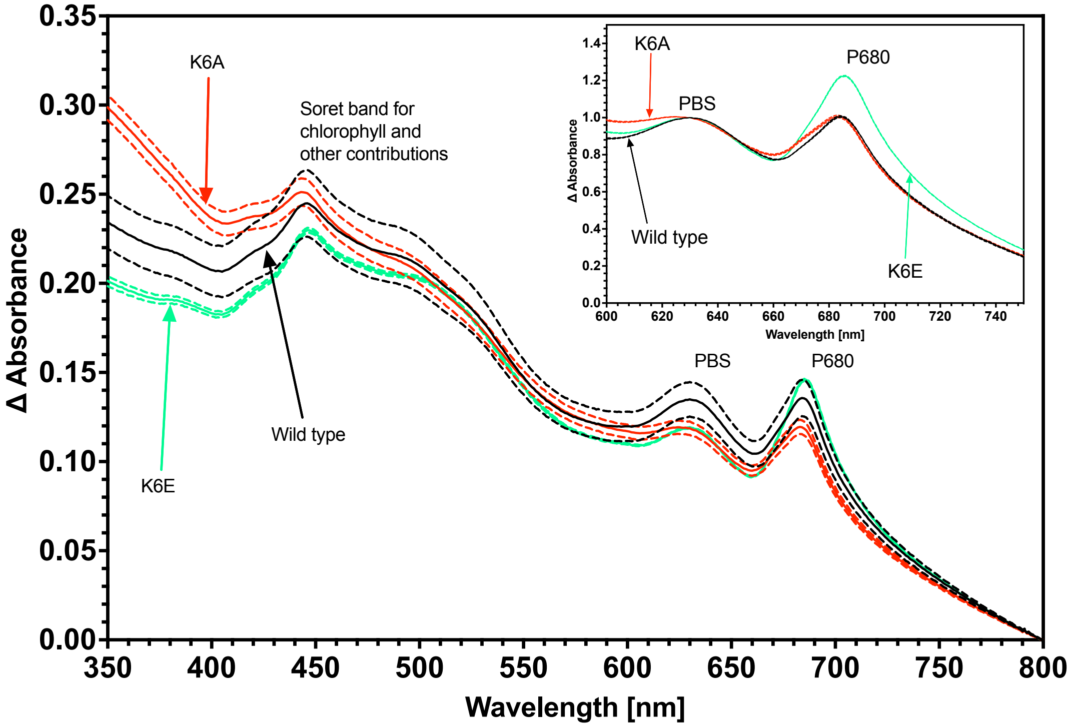

Supplementary Figure 16. Plot of change in absorption compared to 800 nm against wavelength for *Synechocystis* sp. PCC 6803 wild type, K6A, and K6E cells. The major peaks for P680 and PBS absorption and the Soret band for chlorophyll (with additional contributions are labelled). The solid lines represent the mean and the dotted line the 95% C.I. for six experiments. Inset: Plot of change in absorption compared to 630 nm. Source data are provided as a Source Data file.

Supplementary Table 1. MS/MS ions for Figure 1B not labelled in the Figure panel.

**Ion type**	***m/z***	**Ion type**	***m/z***
ay[8-9]	158.09	ay[2-3]	173.13
ay[5-6]	173.13	ay[11-12]	173.09
ay[12-13]	173.09	by[2-3]	201.12
by[5-6]	201.12	by[11-12]	201.09
by[12-13]	201.09	by[9-11]	258.11
ay[10-14]	515.26	ay[8-13]	544.24
by[2-6]	601.36	by[3-7]	601.36
by[5-11]	671.34	by[7-13]	671.30
ay[3-9]	758.44		

Supplementary Table 2. MS/MS ions for Supplementary Figure 2 not labelled in the Figure panel.

**Ion type**	***m/z***	**Ion type**	***m/z***
ay[3-4]	173.13	ay[6-7]	173.13
ay[2-3]	185.17	ay[7-8]	185.17
by[3-4]	201.12	by6-7]	201.12
ay[12-13]	201.12	ay[13-14]	201.12
by[2-3]	213.16	by[7-8]	213.16
by[6-8]	300.19	by[12-14]	300.16
by[2-4]	314.21	a[3]	314.21
ay[4-7]	474.29	by[6-11]	600.30
ay[8-13]	600.30	by[9-14]	600.26
ay[3-9]	786.47	by[4-10]	787.44
ay[4-11]	873.49		

Supplementary Table 3. MS/MS ions for Figure 1C not labelled in the Figure panel.

**Ion type**	***m/z***	**Ion type**	***m/z***
ay[3-4]	173.13	ay[6-7]	173.13
ay[12-13]	173.09	ay[13-14]	173.09
ay[2-3]	185.17	ay[7-8]	185.17
by[3-4]	201.12	by[6-7]	201.12
by[12-13]	201.09	by[13-14]	201.09
by[8-9]	214.12	by[8-10]	285.16
by[6-8]	300.19	by[2-4]	314.21
a[3]	314.20	by[8-11]	400.18
ay[12-15]	400.23	by[6-9]	414.24
by[6-10]	485.27	ay[7-11]	485.27
ay[9-14]	544.24	(parent-2H_2O)$^{+3}$	551.29

Supplementary Table 4. MS/MS ions for Supplementary Figure 4A. The table provides details of b (blue), y (red), and unobserved (black) ions.

No.	b	b^{-18}	b^{-17}	^{+2}b	Peptide sequence	y	y^{-18}	y^{-17}	^{+2}y	No.
1	88.04	70.03	71.01	44.52	S					15
2	201.12	183.11	184.10	101.06	I	1486.82	1468.81	1469.80	743.92	14
3	300.19	282.18	283.17	150.60	V	1373.74	1355.73	1356.71	687.37	13
4	401.24	383.23	384.21	201.12	T	1274.67	1256.66	1257.64	637.83	12
5	529.33	511.32	512.31	265.17	K	1173.62	1155.61	1156.60	587.31	11
6	616.36	598.35	599.38	308.68	S	1045.53	1027.54	1028.51	523.26	10
7	729.45	711.44	712.42	365.23	I	958.49	940.49	941.46	479.75	9
8	828.53	810.50	811.49	414.76	V	845.42	827.46	828.37	423.21	8
9	942.54	924.52	925.54	471.78	N	746.34	728.33	729.31	373.67	7
10	1013.60	995.60	996.58	507.30	A	632.30	614.29	615.27	316.65	6
11	1128.62	1110.56	1111.60	564.81	D	561.26	543.25	544.24	281.14	5
12	1199.69	1181.65	1182.65	600.41	A	446.23	428.29	429.21	223.62	4
13	1328.71	1310.70	1311.68	664.85	E	375.20	357.19	358.17	188.10	3
14	1399.74	1381.73	1382.72	700.37	A	246.16	228.17	229.13	123.58	2
15					R	175.12	158.08	158.09	88.06	1

Supplementary Table 5. MS/MS ions for Supplementary Figure 4B. The table provides details of b (blue), y (red), and unobserved (black) ions.

No.	b	b^{-18}	b^{-17}	^{+2}b	Peptide sequence	y	y^{-18}	y^{-17}	^{+2}y	No.
1	88.04	70.03	71.01	44.52	S					15
2	201.12	183.11	184.10	101.06	I	1558.84	1540.83	1541.82	779.72	14
3	300.19	282.18	283.17	150.60	V	1445.79	1427.70	1428.73	723.38	13
4	401.24	383.23	384.21	201.12	T	1346.69	1328.68	1329.76	673.85	12
5	601.36	583.34	584.33	301.18	K(+72.02)	1245.65	1227.66	1228.62	623.32	11
6	668.42	670.38	671.36	344.69	S	1045.53	1027.54	1028.51	523.26	10
7	801.47	783.46	784.45	401.24	I	958.49	940.49	941.46	479.75	9
8	900.53	882.53	883.51	450.77	V	845.42	827.46	828.37	423.21	8
9	1014.58	996.57	997.56	507.79	N	746.34	728.33	729.31	373.67	7
10	1085.62	1067.61	1068.59	543.31	A	632.30	614.29	615.27	316.65	6
11	1200.65	1182.64	1183.62	600.82	D	561.26	543.25	544.24	281.14	5
12	1271.68	1253.67	1254.66	636.34	A	446.23	428.29	429.21	223.62	4
13	1400.76	1382.72	1383.70	700.86	E	375.20	357.19	358.17	188.10	3
14	1471.76	1453.75	1454.74	736.38	A	246.16	228.17	229.13	123.58	2
15					R	175.12	158.08	158.09	88.06	1

Supplementary Table 6. MS/MS ions for Supplementary Figure 4C. The table provides details of b (blue), y (red), and unobserved (black) ions.

No.	b	b^{-18}	b^{-17}	^{+2}b	Peptide sequence	y	y^{-18}	y^{-17}	^{+2}y	No.
1	116.11	98.06	99.04	58.54	S(+28.03)					15
2	229.15	211.14	212.13	115.08	I	1514.85	1496.84	1497.83	757.95	14
3	328.22	310.21	311.20	164.61	V	1401.78	1383.76	1384.75	701.38	13
4	429.27	411.26	412.24	215.14	T	1302.70	1284.69	1285.67	651.85	12
5	585.39	567.39	568.37	293.20	K(+28.03)	1201.65	1183.64	1184.68	601.33	11
6	672.43	654.41	655.42	336.71	S	1045.53	1027.54	1028.51	523.26	10
7	785.51	767.50	768.49	393.28	I	958.49	940.49	941.46	479.75	9
8	884.58	866.55	867.56	442.82	V	845.42	827.46	828.37	423.21	8
9	998.62	980.61	981.62	499.81	N	746.34	728.33	729.31	373.67	7
10	1069.64	1051.65	1052.66	535.33	A	632.30	614.29	615.27	316.65	6
11	1184.68	1166.68	1167.67	592.84	D	561.26	543.25	544.24	281.14	5
12	1255.74	1237.72	1238.70	628.36	A	446.23	428.29	429.21	223.62	4
13	1384.75	1366.76	1367.74	692.88	E	375.20	357.19	358.17	188.10	3
14	1455.81	1437.80	1438.78	728.40	A	246.16	228.17	229.13	123.58	2
15					R	175.12	157.13	158.09	88.06	1

Supplementary Table 7. Fluorescence lifetimes of ApcAB $(\alpha\beta)_3$ trimers detected at 660 nm (mean±S.D, *n* = 6).

	NaCl				CO_2/HCO_3^-			
	τ_1 (*ns*)	**Yield (%)**	τ_2 (*ns*)	**Yield (%)**	τ_1 (*ns*)	**Yield (%)**	τ_2 (*ns*)	**Yield (%)**
**ApcAB^{WT}**	1.87 ± 0.04	96.8 ± 2.8	0.3 ± 0.1	3.2 ± 2.8	1.86 ± 0.17	98.9 ± 1.0	0.14 ± 0.10	1.07 ± 1.00
**ApcAB^{K6A}**	1.96 ± 0.10	97.0 ± 3.3	0.3 ± 0.2	3.0 ± 3.3	1.91 ± 0.11	95.6 ± 5.3	0.30 ± 0.34	4.43 ± 5.31

Supplementary Table 8. Rotamer states for the ApcA Lys6 in ApcAB $(\alpha\beta)_3$ trimers in the apo and CO_2-bound form. The table shows the occupancy of the different rotamer states for the CO_2-binding Lys6 of ApcA averaged over the three ApcAB $(\alpha\beta)$ monomers.

**Occupied [%]**	**g+**	**a+**	**t**	**a-**	**g-**	**c**
Apo	0.6	0.2	47.0	5.4	46.8	0.0
CO_2	48.9	1.3	36.0	0.1	13.5	0.1
