# Supplementary material for: Allophycocyanin A is a carbon dioxide receptor in the cyanobacterial phycobilisome
Source: Nat Commun. 2022 Sep 8;13:5289. doi: 10.1038/s41467-022-32925-6 (PMC9458709; doi:10.1038/s41467-022-32925-6)
Supplement: Supplementary file 3 — Source Data [file 41467_2022_32925_MOESM3_ESM.zip › Source Data/Source Data_Molecular_Dynamics_parameter.docx]

**SUPPLEMENTARY INFORMATION: PARAMETERS OF NON-STANDARD RESIDUES**

**N-methyl asparagine (MEN)**

***MEN.frcmod***

Remark line goes here

MASS

BOND

C - n2 490.0 1.335

c1 - N 490.0 1.335

ANGLE

c1-c2-n2 94.895 115.295 Calculated with empirical approach for c1-c2-n2

c1-c2-n 90.715 115.000 Calculated with empirical approach for c1-c2-n

c1-c2-o 95.234 119.230 Calculated with empirical approach for c1-c2-o

c1-n -c2 68.100 118.880 same as c1-n -ca, penalty score= 2.8

n -c2-o 115.166 117.460 Calculated with empirical approach for n-c2-o

c1-n -hn 51.835 110.320 Calculated with empirical approach for c1-n-hn

O - C - n2 80.0 122.90 # same as N -C -O 80.0 122.90 AA general

C - n2 - c2 50.0 121.90 # same as C -N -CX 50.0 121.90 AA general (was C-N-CT)

C - n2 - hn 50.0 120.00 # same as C -N -H 50.0 120.00 AA general, gln, asn,changed based on NMA nmodes

CX - C - n2 70.0 116.60 # same as CX-C -N 70.0 116.60 AA general (was CT-C-N)

o - c1 - N 80.0 122.90 # same as N -C -O 80.0 122.90 AA general

c1 - N - H 50.0 120.00 # same as C -N -H 50.0 120.00 AA general, gln, asn,changed based on NMA nmodes

c1 - N - CX 50.0 121.90 # same as C -N -CX 50.0 121.90 AA general (was C-N-CT)

c2 - c1 - N 70.0 116.60 # same as CX-C -N 70.0 116.60 AA general (was CT-C-N)

DIHE

O-C-n2-c2 4 10.00 180.0 2. # same as (O-C-N-CA); X -C -N -X 4 10.00 180.0 2. AA,NMA

CX-C-n2-c2 4 10.00 180.0 2. # same as (CX-C-N-CX); X -C -N -X 4 10.00 180.0 2. AA,NMA

o-c1-N-H 1 2.50 180.0 -2. # same as (O-C-N-H); H -N -C -O 1 2.50 180.0 -2. JCC,7,(1986),230

o-c1-N-H 1 2.00 0.0 1. # same as (O-C-N-H); H -N -C -O 1 2.00 0.0 1. J.C.cistrans-NMA DE

o-c1-N-CX 4 10.00 180.0 2. # same as (O-C-N-CA); X -C -N -X 4 10.00 180.0 2. AA,NMA

c2-c1-N-H 4 10.00 180.0 2. # same as (CX-C-N-H); X -C -N -X 4 10.00 180.0 2. AA,NMA

c2-c1-N-CX 4 10.00 180.0 2. # same as (CA-C-N-CX); X -C -N -X 4 10.00 180.0 2. AA,NMA

O-C-n2-hn 1 2.50 180.0 -2. # same as (O-C-N-H); H -N -C -O 1 2.50 180.0 -2. JCC,7,(1986),230

O-C-n2-hn 1 2.00 0.0 1. # same as (O-C-N-H); H -N -C -O 1 2.00 0.0 1. J.C.cistrans-NMA DE

CX-C-n2-hn 4 10.00 180.0 2. # same as (CX-C-N-H); X -C -N -X 4 10.00 180.0 2. AA,NMA

IMPROPER

c1-c1-c2-n2 1.1 180.0 2.0 Using the default value

c1-n -c2-o 1.1 180.0 2.0 Using the default value

c1-c2-n -hn 1.1 180.0 2.0 Using the default value

NONBON

***MEN.lib***

!!index array str

"MEN"

!entry.MEN.unit.atoms table str name str type int typex int resx int flags int seq int elmnt dbl chg

"N" "n2" 0 1 131072 1 7 -0.314000

"CA" "c2" 0 1 131072 2 6 0.395000

"C" "c1" 0 1 131072 3 6 0.165000

"O" "o" 0 1 131072 4 8 -0.153000

"CB" "c1" 0 1 131072 5 6 -0.655000

"CG" "c2" 0 1 131072 6 6 0.442000

"ND2" "n" 0 1 131072 7 7 -0.388000

"OD1" "o" 0 1 131072 8 8 -0.193000

"CE2" "c1" 0 1 131072 9 6 0.095000

"H" "hn" 0 1 131072 10 1 0.319000

"H1" "hn" 0 1 131072 11 1 0.288000

!entry.MEN.unit.atomspertinfo table str pname str ptype int ptypex int pelmnt dbl pchg

"N" "n2" 0 -1 0.0

"CA" "c2" 0 -1 0.0

"C" "c1" 0 -1 0.0

"O" "o" 0 -1 0.0

"CB" "c1" 0 -1 0.0

"CG" "c2" 0 -1 0.0

"ND2" "n" 0 -1 0.0

"OD1" "o" 0 -1 0.0

"CE2" "c1" 0 -1 0.0

"H" "hn" 0 -1 0.0

"H1" "hn" 0 -1 0.0

!entry.MEN.unit.boundbox array dbl

-1.000000

0.0

0.0

0.0

0.0

!entry.MEN.unit.childsequence single int

2

!entry.MEN.unit.connect array int

1

3

!entry.MEN.unit.connectivity table int atom1x int atom2x int flags

1 2 1

1 10 1

2 3 1

2 5 1

3 4 1

5 6 1

6 7 1

6 8 1

7 9 1

7 11 1

!entry.MEN.unit.hierarchy table str abovetype int abovex str belowtype int belowx

"U" 0 "R" 1

"R" 1 "A" 1

"R" 1 "A" 2

"R" 1 "A" 3

"R" 1 "A" 4

"R" 1 "A" 5

"R" 1 "A" 6

"R" 1 "A" 7

"R" 1 "A" 8

"R" 1 "A" 9

"R" 1 "A" 10

"R" 1 "A" 11

!entry.MEN.unit.name single str

"MEN"

!entry.MEN.unit.positions table dbl x dbl y dbl z

-6.226000 -27.169000 33.235000

-5.573000 -25.955000 32.760000

-4.669000 -26.202000 31.556000

-4.169000 -25.260000 30.941000

-4.700000 -25.350000 33.861000

-3.599000 -24.465000 33.311000

-3.968000 -23.531000 32.443000

-2.429000 -24.619000 33.663000

-3.865000 -22.063000 31.863000

-6.803000 -27.559000 32.490000

-4.643000 -23.972000 31.878000

!entry.MEN.unit.residueconnect table int c1x int c2x int c3x int c4x int c5x int c6x

0 0 0 0 0 0

!entry.MEN.unit.residues table str name int seq int childseq int startatomx str restype int imagingx

"MEN" 1 12 1 "?" 0

!entry.MEN.unit.residuesPdbSequenceNumber array int

0

!entry.MEN.unit.solventcap array dbl

-1.000000

0.0

0.0

0.0

0.0

!entry.MEN.unit.velocities table dbl x dbl y dbl z

0.0 0.0 0.0

0.0 0.0 0.0

0.0 0.0 0.0

0.0 0.0 0.0

0.0 0.0 0.0

0.0 0.0 0.0

0.0 0.0 0.0

0.0 0.0 0.0

0.0 0.0 0.0

0.0 0.0 0.0

0.0 0.0 0.0

**CO_2_-bound Lysine (LYE)**

***LYE.frcmod***

Remark line goes here

MASS

BOND

C - n2 490.0 1.335

c1 - N 490.0 1.335

ANGLE

c1-c3-n2 85.814 109.895 Calculated with empirical approach for c1-c3-n2

c3-c1-o 72.683 180.000 Calculated with empirical approach for c3-c1-o

n -c2-o 115.166 117.460 Calculated with empirical approach for n-c2-o

C - n2 - c3 50.0 121.90 # same as C -N -CX 50.0 121.90 AA general (was C-N-CT)

c3 - c1 - N 70.0 116.60 # same as CX-C -N 70.0 116.60 AA general (was CT-C-N)

DIHE

O-C-n2-c3 4 10.00 180.0 2. # same as (O-C-N-CA); X -C -N -X 4 10.00 180.0 2. AA,NMA

CX-C-n2-c3 4 10.00 180.0 2. # same as (CX-C-N-CX); X -C -N -X 4 10.00 180.0 2. AA,NMA

c3-c1-N-H 4 10.00 180.0 2. # same as (CX-C-N-H); X -C -N -X 4 10.00 180.0 2. AA,NMA

c3-c1-N-CX 4 10.00 180.0 2. # same as (CA-C-N-CX); X -C -N -X 4 10.00 180.0 2. AA,NMA

IMPROPER

c2-c3-n -hn 1.1 180.0 2.0 Using the default value

n -o -c2-o 1.1 180.0 2.0 Using the default value

NONBON

***LYE.lib***

!!index array str

"LYE"

!entry.LYE.unit.atoms table str name str type int typex int resx int flags int seq int elmnt dbl chg

"N" "n2" 0 1 131072 1 7 -0.291000

"CA" "c3" 0 1 131072 2 6 -0.037000

"C03" "c3" 0 1 131072 3 6 -0.147000

"C04" "c3" 0 1 131072 4 6 -0.158000

"C05" "c3" 0 1 131072 5 6 -0.162000

"C06" "c3" 0 1 131072 6 6 -0.028000

"N07" "n" 0 1 131072 7 7 -0.413000

"C08" "c2" 0 1 131072 8 6 0.391000

"O09" "o" 0 1 131072 9 8 -0.579500

"O10" "o" 0 1 131072 10 8 -0.579500

"C" "c1" 0 1 131072 11 6 0.219000

"O" "o" 0 1 131072 12 8 -0.221000

"H012" "hn" 0 1 131072 13 1 0.129000

"H021" "h1" 0 1 131072 14 1 0.095000

"H031" "hc" 0 1 131072 15 1 0.095500

"H032" "hc" 0 1 131072 16 1 0.095500

"H041" "hc" 0 1 131072 17 1 0.101500

"H042" "hc" 0 1 131072 18 1 0.101500

"H051" "hc" 0 1 131072 19 1 0.054000

"H052" "hc" 0 1 131072 20 1 0.054000

"H061" "h1" 0 1 131072 21 1 0.061500

"H062" "h1" 0 1 131072 22 1 0.061500

"H071" "hn" 0 1 131072 23 1 0.155000

!entry.LYE.unit.atomspertinfo table str pname str ptype int ptypex int pelmnt dbl pchg

"N" "n2" 0 -1 0.0

"CA" "c3" 0 -1 0.0

"C03" "c3" 0 -1 0.0

"C04" "c3" 0 -1 0.0

"C05" "c3" 0 -1 0.0

"C06" "c3" 0 -1 0.0

"N07" "n" 0 -1 0.0

"C08" "c2" 0 -1 0.0

"O09" "o" 0 -1 0.0

"O10" "o" 0 -1 0.0

"C" "c1" 0 -1 0.0

"O" "o" 0 -1 0.0

"H012" "hn" 0 -1 0.0

"H021" "h1" 0 -1 0.0

"H031" "hc" 0 -1 0.0

"H032" "hc" 0 -1 0.0

"H041" "hc" 0 -1 0.0

"H042" "hc" 0 -1 0.0

"H051" "hc" 0 -1 0.0

"H052" "hc" 0 -1 0.0

"H061" "h1" 0 -1 0.0

"H062" "h1" 0 -1 0.0

"H071" "hn" 0 -1 0.0

!entry.LYE.unit.boundbox array dbl

-1.000000

0.0

0.0

0.0

0.0

!entry.LYE.unit.childsequence single int

2

!entry.LYE.unit.connect array int

1

11

!entry.LYE.unit.connectivity table int atom1x int atom2x int flags

1 2 1

1 13 1

2 3 1

2 11 1

2 14 1

3 4 1

3 15 1

3 16 1

4 5 1

4 17 1

4 18 1

5 6 1

5 19 1

5 20 1

6 7 1

6 21 1

6 22 1

7 8 1

7 23 1

8 9 1

8 10 1

11 12 1

!entry.LYE.unit.hierarchy table str abovetype int abovex str belowtype int belowx

"U" 0 "R" 1

"R" 1 "A" 1

"R" 1 "A" 2

"R" 1 "A" 3

"R" 1 "A" 4

"R" 1 "A" 5

"R" 1 "A" 6

"R" 1 "A" 7

"R" 1 "A" 8

"R" 1 "A" 9

"R" 1 "A" 10

"R" 1 "A" 11

"R" 1 "A" 12

"R" 1 "A" 13

"R" 1 "A" 14

"R" 1 "A" 15

"R" 1 "A" 16

"R" 1 "A" 17

"R" 1 "A" 18

"R" 1 "A" 19

"R" 1 "A" 20

"R" 1 "A" 21

"R" 1 "A" 22

"R" 1 "A" 23

!entry.LYE.unit.name single str

"LYE"

!entry.LYE.unit.positions table dbl x dbl y dbl z

25.754000 -12.021000 17.614000

24.901000 -11.685000 16.471000

24.121000 -10.389000 16.712000

24.262000 -9.401000 15.556000

24.979000 -8.119000 15.973000

25.271000 -7.206000 14.784000

24.091000 -7.071000 13.949000

24.219000 -7.080000 12.502000

23.720000 -6.148000 11.820000

24.829000 -8.022000 11.931000

24.027000 -12.839000 15.972000

22.818000 -12.640000 15.681000

26.578000 -12.267000 16.795000

25.511000 -11.489000 15.742000

23.182000 -10.605000 16.827000

24.449000 -9.972000 17.523000

23.378000 -9.174000 15.227000

24.764000 -9.823000 14.841000

24.423000 -7.640000 16.607000

25.816000 -8.353000 16.403000

25.993000 -7.586000 14.259000

25.536000 -6.332000 15.109000

23.319000 -6.953000 14.310000

!entry.LYE.unit.residueconnect table int c1x int c2x int c3x int c4x int c5x int c6x

0 0 0 0 0 0

!entry.LYE.unit.residues table str name int seq int childseq int startatomx str restype int imagingx

"LYE" 1 24 1 "?" 0

!entry.LYE.unit.residuesPdbSequenceNumber array int

0

!entry.LYE.unit.solventcap array dbl

-1.000000

0.0

0.0

0.0

0.0

!entry.LYE.unit.velocities table dbl x dbl y dbl z

0.0 0.0 0.0

0.0 0.0 0.0

0.0 0.0 0.0

0.0 0.0 0.0

0.0 0.0 0.0

0.0 0.0 0.0

0.0 0.0 0.0

0.0 0.0 0.0

0.0 0.0 0.0

0.0 0.0 0.0

0.0 0.0 0.0

0.0 0.0 0.0

0.0 0.0 0.0

0.0 0.0 0.0

0.0 0.0 0.0

0.0 0.0 0.0

0.0 0.0 0.0

0.0 0.0 0.0

0.0 0.0 0.0

0.0 0.0 0.0

0.0 0.0 0.0

0.0 0.0 0.0

0.0 0.0 0.0

**Chromophore (CYC)**

***CYC.frcmod***

Remark line goes here

MASS

BOND

ANGLE

ce-c2-n 87.800 120.710 same as ce-c2-nh, penalty score= 1.8

DIHE

nd-cd-ce-cc 4 16.000 180.000 2.000 same as X -cc-cd-X , penalty score=136.0

nd-cd-ce-ha 4 16.000 180.000 2.000 same as X -cc-cd-X , penalty score=136.0

nd-cc-ce-cd 4 4.000 180.000 2.000 same as X -ce-ce-X , penalty score=136.0

nd-cc-ce-ha 4 4.000 180.000 2.000 same as X -ce-ce-X , penalty score=136.0

na-cc-ce-cd 4 4.000 180.000 2.000 same as X -ce-ce-X , penalty score=136.0

cd-cc-ce-cd 1 0.500 180.000 2.000 same as c2-ce-ce-c2, penalty score=310.0

cd-cd-ce-cc 1 8.510 180.000 2.000 same as ca-cf-ce-ca, penalty score=310.0

cd-cd-ce-ha 4 16.000 180.000 2.000 same as X -cc-cd-X , penalty score=136.0

cc-cc-ce-cd 1 0.500 180.000 2.000 same as c2-ce-ce-c2, penalty score=310.0

cc-cc-ce-ha 4 4.000 180.000 2.000 same as X -ce-ce-X , penalty score=136.0

n -cd-ce-cc 4 26.600 180.000 2.000 same as X -ce-cf-X , penalty score=136.0

n -cd-ce-ha 4 16.000 180.000 2.000 same as X -cc-cd-X , penalty score=136.0

na-cc-ce-c2 4 4.000 180.000 2.000 same as X -ce-ce-X , penalty score=136.0

cd-cc-ce-c2 1 0.500 180.000 2.000 same as c2-ce-ce-c2, penalty score=223.0

na-cc-ce-ha 4 4.000 180.000 2.000 same as X -ce-ce-X , penalty score=136.0

cd-cc-ce-ha 4 4.000 180.000 2.000 same as X -ce-ce-X , penalty score=136.0

IMPROPER

cc-cd-ce-ha 1.1 180.0 2.0 Same as X -X -ca-ha, penalty score= 46.8 (use general term))

cd-ce-cd-nd 1.1 180.0 2.0 Using the default value

c3-cc-cd-cd 1.1 180.0 2.0 Using the default value

c3-cc-cc-cd 1.1 180.0 2.0 Using the default value

cc-ce-cc-nd 1.1 180.0 2.0 Using the default value

c -cd-n -hn 1.1 180.0 2.0 Using general improper torsional angle X- X- n-hn, penalty score= 6.0)

cd-ce-cd-n 1.1 180.0 2.0 Using the default value

c -c3-cc-cd 1.1 180.0 2.0 Using the default value

cc-n -c -o 10.5 180.0 2.0 Using general improper torsional angle X- X- c- o, penalty score= 6.0)

c -c2-n -hn 1.1 180.0 2.0 Using general improper torsional angle X- X- n-hn, penalty score= 6.0)

c3-n -c -o 10.5 180.0 2.0 Using general improper torsional angle X- X- c- o, penalty score= 6.0)

c3-ce-c2-n 1.1 180.0 2.0 Using the default value

c2-cc-ce-ha 1.1 180.0 2.0 Same as X -X -ca-ha, penalty score= 46.8 (use general term))

cc-cc-na-hn 1.1 180.0 2.0 Using general improper torsional angle X- X-na-hn, penalty score= 6.0)

cd-ce-cc-na 1.1 180.0 2.0 Using the default value

NONBON

***CYC.lib***

!!index array str

"CYC"

!entry.CYC.unit.atoms table str name str type int typex int resx int flags int seq int elmnt dbl chg

"CHA" "ce" 0 1 131072 1 6 0.038800

"NA" "nd" 0 1 131072 2 7 -0.576300

"C1A" "cd" 0 1 131072 3 6 0.029600

"C2A" "cd" 0 1 131072 4 6 -0.092200

"C3A" "cc" 0 1 131072 5 6 -0.228800

"C4A" "cc" 0 1 131072 6 6 0.478900

"CMA" "c3" 0 1 131072 7 6 -0.042900

"CAA" "c3" 0 1 131072 8 6 -0.025200

"CBA" "c3" 0 1 131072 9 6 -0.121400

"CGA" "c" 0 1 131072 10 6 0.630100

"O1A" "o" 0 1 131072 11 8 -0.546000

"O2A" "oh" 0 1 131072 12 8 -0.625100

"CHB" "ce" 0 1 131072 13 6 -0.240600

"NB" "n" 0 1 131072 14 7 -0.507100

"C1B" "cd" 0 1 131072 15 6 0.068600

"C2B" "cd" 0 1 131072 16 6 -0.052200

"C3B" "cc" 0 1 131072 17 6 -0.153400

"C4B" "c" 0 1 131072 18 6 0.698700

"CMB" "c3" 0 1 131072 19 6 -0.054900

"CAB" "c3" 0 1 131072 20 6 -0.022200

"CBB" "c3" 0 1 131072 21 6 -0.095100

"OB" "o" 0 1 131072 22 8 -0.593500

"NC" "n" 0 1 131072 23 7 -0.489100

"C1C" "c" 0 1 131072 24 6 0.699500

"C2C" "c3" 0 1 131072 25 6 -0.137700

"C3C" "c3" 0 1 131072 26 6 -0.037500

"C4C" "c2" 0 1 131072 27 6 0.062400

"CMC" "c3" 0 1 131072 28 6 -0.087100

"CAC" "c3" 0 1 131072 29 6 -0.075400

"CBC" "c3" 0 1 131072 30 6 -0.097100

"OC" "o" 0 1 131072 31 8 -0.599500

"CHD" "ce" 0 1 131072 32 6 -0.202200

"ND" "na" 0 1 131072 33 7 -0.123100

"C1D" "cc" 0 1 131072 34 6 -0.037100

"C2D" "cd" 0 1 131072 35 6 -0.153300

"C3D" "cd" 0 1 131072 36 6 -0.106300

"C4D" "cc" 0 1 131072 37 6 -0.118100

"CMD" "c3" 0 1 131072 38 6 -0.027800

"CAD" "c3" 0 1 131072 39 6 -0.008100

"CBD" "c3" 0 1 131072 40 6 -0.125400

"CGD" "c" 0 1 131072 41 6 0.637100

"O1D" "o" 0 1 131072 42 8 -0.541000

"O2D" "oh" 0 1 131072 43 8 -0.631100

"H" "ha" 0 1 131072 44 1 0.157000

"H1" "hc" 0 1 131072 45 1 0.049033

"H2" "hc" 0 1 131072 46 1 0.049033

"H3" "hc" 0 1 131072 47 1 0.049033

"H4" "hc" 0 1 131072 48 1 0.068200

"H5" "hc" 0 1 131072 49 1 0.068200

"H6" "hc" 0 1 131072 50 1 0.085200

"H7" "hc" 0 1 131072 51 1 0.085200

"H8" "ho" 0 1 131072 52 1 0.461000

"H9" "ha" 0 1 131072 53 1 0.157000

"H10" "hn" 0 1 131072 54 1 0.347500

"H11" "hc" 0 1 131072 55 1 0.056700

"H12" "hc" 0 1 131072 56 1 0.056700

"H13" "hc" 0 1 131072 57 1 0.056700

"H14" "hc" 0 1 131072 58 1 0.056200

"H15" "hc" 0 1 131072 59 1 0.056200

"H16" "hc" 0 1 131072 60 1 0.043700

"H17" "hc" 0 1 131072 61 1 0.043700

"H18" "hc" 0 1 131072 62 1 0.043700

"H19" "hn" 0 1 131072 63 1 0.348500

"H20" "hc" 0 1 131072 64 1 0.090700

"H21" "hc" 0 1 131072 65 1 0.078700

"H22" "hc" 0 1 131072 66 1 0.049700

"H23" "hc" 0 1 131072 67 1 0.049700

"H24" "hc" 0 1 131072 68 1 0.049700

"H25" "hc" 0 1 131072 69 1 0.051700

"H26" "hc" 0 1 131072 70 1 0.051700

"H27" "hc" 0 1 131072 71 1 0.038033

"H28" "hc" 0 1 131072 72 1 0.038033

"H29" "hc" 0 1 131072 73 1 0.038033

"H30" "ha" 0 1 131072 74 1 0.149000

"H31" "hn" 0 1 131072 75 1 0.326700

"H32" "hc" 0 1 131072 76 1 0.043700

"H33" "hc" 0 1 131072 77 1 0.043700

"H34" "hc" 0 1 131072 78 1 0.043700

"H35" "hc" 0 1 131072 79 1 0.062700

"H36" "hc" 0 1 131072 80 1 0.062700

"H37" "hc" 0 1 131072 81 1 0.086200

"H38" "hc" 0 1 131072 82 1 0.086200

"H39" "ho" 0 1 131072 83 1 0.449000

!entry.CYC.unit.atomspertinfo table str pname str ptype int ptypex int pelmnt dbl pchg

"CHA" "ce" 0 -1 0.0

"NA" "nd" 0 -1 0.0

"C1A" "cd" 0 -1 0.0

"C2A" "cd" 0 -1 0.0

"C3A" "cc" 0 -1 0.0

"C4A" "cc" 0 -1 0.0

"CMA" "c3" 0 -1 0.0

"CAA" "c3" 0 -1 0.0

"CBA" "c3" 0 -1 0.0

"CGA" "c" 0 -1 0.0

"O1A" "o" 0 -1 0.0

"O2A" "oh" 0 -1 0.0

"CHB" "ce" 0 -1 0.0

"NB" "n" 0 -1 0.0

"C1B" "cd" 0 -1 0.0

"C2B" "cd" 0 -1 0.0

"C3B" "cc" 0 -1 0.0

"C4B" "c" 0 -1 0.0

"CMB" "c3" 0 -1 0.0

"CAB" "c3" 0 -1 0.0

"CBB" "c3" 0 -1 0.0

"OB" "o" 0 -1 0.0

"NC" "n" 0 -1 0.0

"C1C" "c" 0 -1 0.0

"C2C" "c3" 0 -1 0.0

"C3C" "c3" 0 -1 0.0

"C4C" "c2" 0 -1 0.0

"CMC" "c3" 0 -1 0.0

"CAC" "c3" 0 -1 0.0

"CBC" "c3" 0 -1 0.0

"OC" "o" 0 -1 0.0

"CHD" "ce" 0 -1 0.0

"ND" "na" 0 -1 0.0

"C1D" "cc" 0 -1 0.0

"C2D" "cd" 0 -1 0.0

"C3D" "cd" 0 -1 0.0

"C4D" "cc" 0 -1 0.0

"CMD" "c3" 0 -1 0.0

"CAD" "c3" 0 -1 0.0

"CBD" "c3" 0 -1 0.0

"CGD" "c" 0 -1 0.0

"O1D" "o" 0 -1 0.0

"O2D" "oh" 0 -1 0.0

"H" "ha" 0 -1 0.0

"H1" "hc" 0 -1 0.0

"H2" "hc" 0 -1 0.0

"H3" "hc" 0 -1 0.0

"H4" "hc" 0 -1 0.0

"H5" "hc" 0 -1 0.0

"H6" "hc" 0 -1 0.0

"H7" "hc" 0 -1 0.0

"H8" "ho" 0 -1 0.0

"H9" "ha" 0 -1 0.0

"H10" "hn" 0 -1 0.0

"H11" "hc" 0 -1 0.0

"H12" "hc" 0 -1 0.0

"H13" "hc" 0 -1 0.0

"H14" "hc" 0 -1 0.0

"H15" "hc" 0 -1 0.0

"H16" "hc" 0 -1 0.0

"H17" "hc" 0 -1 0.0

"H18" "hc" 0 -1 0.0

"H19" "hn" 0 -1 0.0

"H20" "hc" 0 -1 0.0

"H21" "hc" 0 -1 0.0

"H22" "hc" 0 -1 0.0

"H23" "hc" 0 -1 0.0

"H24" "hc" 0 -1 0.0

"H25" "hc" 0 -1 0.0

"H26" "hc" 0 -1 0.0

"H27" "hc" 0 -1 0.0

"H28" "hc" 0 -1 0.0

"H29" "hc" 0 -1 0.0

"H30" "ha" 0 -1 0.0

"H31" "hn" 0 -1 0.0

"H32" "hc" 0 -1 0.0

"H33" "hc" 0 -1 0.0

"H34" "hc" 0 -1 0.0

"H35" "hc" 0 -1 0.0

"H36" "hc" 0 -1 0.0

"H37" "hc" 0 -1 0.0

"H38" "hc" 0 -1 0.0

"H39" "ho" 0 -1 0.0

!entry.CYC.unit.boundbox array dbl

-1.000000

0.0

0.0

0.0

0.0

!entry.CYC.unit.childsequence single int

2

!entry.CYC.unit.connect array int

0

0

!entry.CYC.unit.connectivity table int atom1x int atom2x int flags

1 3 2

1 37 1

1 44 1

2 3 1

2 6 2

3 4 1

4 5 2

4 8 1

5 6 1

5 7 1

6 13 1

7 45 1

7 46 1

7 47 1

8 9 1

8 48 1

8 49 1

9 10 1

9 50 1

9 51 1

10 11 2

10 12 1

12 52 1

13 15 2

13 53 1

14 15 1

14 18 1

14 54 1

15 16 1

16 17 2

16 19 1

17 18 1

17 20 1

18 22 2

19 55 1

19 56 1

19 57 1

20 21 1

20 58 1

20 59 1

21 60 1

21 61 1

21 62 1

23 24 1

23 27 1

23 63 1

24 25 1

24 31 2

25 26 1

25 28 1

25 64 1

26 27 1

26 29 1

26 65 1

27 32 2

28 66 1

28 67 1

28 68 1

29 30 1

29 69 1

29 70 1

30 71 1

30 72 1

30 73 1

32 34 1

32 74 1

33 34 1

33 37 1

33 75 1

34 35 2

35 36 1

35 38 1

36 37 2

36 39 1

38 76 1

38 77 1

38 78 1

39 40 1

39 79 1

39 80 1

40 41 1

40 81 1

40 82 1

41 42 2

41 43 1

43 83 1

!entry.CYC.unit.hierarchy table str abovetype int abovex str belowtype int belowx

"U" 0 "R" 1

"R" 1 "A" 1

"R" 1 "A" 2

"R" 1 "A" 3

"R" 1 "A" 4

"R" 1 "A" 5

"R" 1 "A" 6

"R" 1 "A" 7

"R" 1 "A" 8

"R" 1 "A" 9

"R" 1 "A" 10

"R" 1 "A" 11

"R" 1 "A" 12

"R" 1 "A" 13

"R" 1 "A" 14

"R" 1 "A" 15

"R" 1 "A" 16

"R" 1 "A" 17

"R" 1 "A" 18

"R" 1 "A" 19

"R" 1 "A" 20

"R" 1 "A" 21

"R" 1 "A" 22

"R" 1 "A" 23

"R" 1 "A" 24

"R" 1 "A" 25

"R" 1 "A" 26

"R" 1 "A" 27

"R" 1 "A" 28

"R" 1 "A" 29

"R" 1 "A" 30

"R" 1 "A" 31

"R" 1 "A" 32

"R" 1 "A" 33

"R" 1 "A" 34

"R" 1 "A" 35

"R" 1 "A" 36

"R" 1 "A" 37

"R" 1 "A" 38

"R" 1 "A" 39

"R" 1 "A" 40

"R" 1 "A" 41

"R" 1 "A" 42

"R" 1 "A" 43

"R" 1 "A" 44

"R" 1 "A" 45

"R" 1 "A" 46

"R" 1 "A" 47

"R" 1 "A" 48

"R" 1 "A" 49

"R" 1 "A" 50

"R" 1 "A" 51

"R" 1 "A" 52

"R" 1 "A" 53

"R" 1 "A" 54

"R" 1 "A" 55

"R" 1 "A" 56

"R" 1 "A" 57

"R" 1 "A" 58

"R" 1 "A" 59

"R" 1 "A" 60

"R" 1 "A" 61

"R" 1 "A" 62

"R" 1 "A" 63

"R" 1 "A" 64

"R" 1 "A" 65

"R" 1 "A" 66

"R" 1 "A" 67

"R" 1 "A" 68

"R" 1 "A" 69

"R" 1 "A" 70

"R" 1 "A" 71

"R" 1 "A" 72

"R" 1 "A" 73

"R" 1 "A" 74

"R" 1 "A" 75

"R" 1 "A" 76

"R" 1 "A" 77

"R" 1 "A" 78

"R" 1 "A" 79

"R" 1 "A" 80

"R" 1 "A" 81

"R" 1 "A" 82

"R" 1 "A" 83

!entry.CYC.unit.name single str

"CYC"

!entry.CYC.unit.positions table dbl x dbl y dbl z

16.036000 -11.564000 -17.286000

14.347000 -13.345000 -17.234000

14.816000 -12.063000 -17.035000

13.719000 -11.261000 -16.506000

12.588000 -11.981000 -16.480000

13.085000 -13.309000 -16.887000

11.211000 -11.524000 -16.178000

13.829000 -9.820000 -16.073000

14.278000 -9.684000 -14.613000

15.761000 -9.530000 -14.420000

16.429000 -10.078000 -13.560000

16.315000 -8.606000 -15.227000

12.185000 -14.488000 -16.931000

11.114000 -14.305000 -14.771000

11.310000 -14.876000 -15.993000

10.376000 -15.991000 -16.042000

9.633000 -16.039000 -14.916000

10.119000 -14.930000 -14.056000

10.299000 -16.919000 -17.206000

8.573000 -16.979000 -14.454000

7.190000 -16.343000 -14.407000

9.714000 -14.642000 -12.949000

20.510000 -14.663000 -20.797000

21.357000 -15.294000 -21.670000

20.975000 -16.754000 -21.721000

19.785000 -16.880000 -20.727000

19.611000 -15.473000 -20.173000

20.592000 -17.130000 -23.146000

20.059000 -17.947000 -19.650000

20.032000 -19.359000 -20.225000

22.252000 -14.758000 -22.303000

18.695000 -15.093000 -19.264000

17.241000 -13.451000 -18.295000

18.527000 -13.790000 -18.705000

19.355000 -12.724000 -18.360000

18.522000 -11.709000 -17.778000

17.220000 -12.190000 -17.758000

20.838000 -12.601000 -18.492000

18.986000 -10.385000 -17.235000

19.055000 -9.299000 -18.302000

19.635000 -8.010000 -17.787000

20.076000 -7.109000 -18.482000

19.598000 -7.863000 -16.449000

16.176000 -10.507000 -17.093000

10.450000 -12.269000 -16.417000

11.116000 -11.255000 -15.121000

10.964000 -10.640000 -16.777000

14.455000 -9.245000 -16.764000

12.840000 -9.352000 -16.150000

13.813000 -8.798000 -14.165000

13.949000 -10.549000 -14.023000

17.254000 -8.567000 -14.939000

12.313000 -15.077000 -17.834000

11.634000 -13.537000 -14.377000

9.565000 -17.715000 -17.057000

10.011000 -16.376000 -18.113000

11.268000 -17.399000 -17.379000

8.528000 -17.858000 -15.106000

8.830000 -17.357000 -13.457000

6.439000 -17.096000 -14.143000

7.140000 -15.548000 -13.656000

6.912000 -15.917000 -15.377000

20.556000 -13.665000 -20.672000

21.842000 -17.341000 -21.397000

18.868000 -17.148000 -21.268000

20.275000 -18.175000 -23.205000

19.777000 -16.500000 -23.519000

21.442000 -16.994000 -23.825000

21.030000 -17.770000 -19.171000

19.304000 -17.890000 -18.858000

20.183000 -20.095000 -19.429000

19.066000 -19.568000 -20.698000

20.818000 -19.509000 -20.972000

18.004000 -15.858000 -18.913000

16.419000 -14.038000 -18.372000

21.091000 -12.087000 -19.426000

21.266000 -12.012000 -17.676000

21.333000 -13.575000 -18.465000

18.315000 -10.071000 -16.429000

19.963000 -10.505000 -16.753000

19.692000 -9.634000 -19.130000

18.058000 -9.085000 -18.701000

20.040000 -6.997000 -16.309000

!entry.CYC.unit.residueconnect table int c1x int c2x int c3x int c4x int c5x int c6x

0 0 0 0 0 0

!entry.CYC.unit.residues table str name int seq int childseq int startatomx str restype int imagingx

"CYC" 1 84 1 "?" 0

!entry.CYC.unit.residuesPdbSequenceNumber array int

0

!entry.CYC.unit.solventcap array dbl

-1.000000

0.0

0.0

0.0

0.0

!entry.CYC.unit.velocities table dbl x dbl y dbl z

0.0 0.0 0.0

0.0 0.0 0.0

0.0 0.0 0.0

0.0 0.0 0.0

0.0 0.0 0.0

0.0 0.0 0.0

0.0 0.0 0.0

0.0 0.0 0.0

0.0 0.0 0.0

0.0 0.0 0.0

0.0 0.0 0.0

0.0 0.0 0.0

0.0 0.0 0.0

0.0 0.0 0.0

0.0 0.0 0.0

0.0 0.0 0.0

0.0 0.0 0.0

0.0 0.0 0.0

0.0 0.0 0.0

0.0 0.0 0.0

0.0 0.0 0.0

0.0 0.0 0.0

0.0 0.0 0.0

0.0 0.0 0.0

0.0 0.0 0.0

0.0 0.0 0.0

0.0 0.0 0.0

0.0 0.0 0.0

0.0 0.0 0.0

0.0 0.0 0.0

0.0 0.0 0.0

0.0 0.0 0.0

0.0 0.0 0.0

0.0 0.0 0.0

0.0 0.0 0.0

0.0 0.0 0.0

0.0 0.0 0.0

0.0 0.0 0.0

0.0 0.0 0.0

0.0 0.0 0.0

0.0 0.0 0.0

0.0 0.0 0.0

0.0 0.0 0.0

0.0 0.0 0.0

0.0 0.0 0.0

0.0 0.0 0.0

0.0 0.0 0.0

0.0 0.0 0.0

0.0 0.0 0.0

0.0 0.0 0.0

0.0 0.0 0.0

0.0 0.0 0.0

0.0 0.0 0.0

0.0 0.0 0.0

0.0 0.0 0.0

0.0 0.0 0.0

0.0 0.0 0.0

0.0 0.0 0.0

0.0 0.0 0.0

0.0 0.0 0.0

0.0 0.0 0.0

0.0 0.0 0.0

0.0 0.0 0.0

0.0 0.0 0.0

0.0 0.0 0.0

0.0 0.0 0.0

0.0 0.0 0.0

0.0 0.0 0.0

0.0 0.0 0.0

0.0 0.0 0.0

0.0 0.0 0.0

0.0 0.0 0.0

0.0 0.0 0.0

0.0 0.0 0.0

0.0 0.0 0.0

0.0 0.0 0.0

0.0 0.0 0.0

0.0 0.0 0.0

0.0 0.0 0.0

0.0 0.0 0.0

0.0 0.0 0.0

0.0 0.0 0.0

0.0 0.0 0.0
